# NSP6 inhibits the production of ACE2-containing exosomes to promote SARS-CoV-2 infectivity

**DOI:** 10.1128/mbio.03358-23

**Published:** 2024-02-02

**Authors:** Xi Lv, Ran Chen, Taizhen Liang, Haojie Peng, Qiannan Fang, Shiqi Xiao, Sen Liu, Meilin Hu, Fei Yu, Lixue Cao, Yiwen Zhang, Ting Pan, Zhihui Xi, Yao Ding, Linyuan Feng, Tao Zeng, Wenjing Huang, Hui Zhang, Xiancai Ma

**Affiliations:** 1 School of Medicine, South China University of Technology, Guangzhou, Guangdong, China; 2 Guangdong Provincial People's Hospital (Guangdong Academy of Medical Sciences), Southern Medical University, Guangzhou, Guangdong, China; 3 Institute of Human Virology, Key Laboratory of Tropical Disease Control of Ministry Education, Guangdong Engineering Research Center for Antimicrobial Agent and Immunotechnology, Zhongshan School of Medicine, Sun Yat-sen University, Guangzhou, Guangdong, China; 4 Guangzhou National Laboratory, Guangzhou International Bio-Island, Guangzhou, Guangdong, China; 5 Department of Breast Surgery, the Second Affiliated Hospital of Guangzhou Medical University, Guangzhou, Guangdong, China; 6 Center for Infection and Immunity Studies, School of Medicine, Shenzhen Campus of Sun Yat-sen University, Shenzhen, Guangdong, China; University of Calgary, Canada

**Keywords:** NSP6, ACE2, exosomes, SARS-CoV-2, CD63, PSMD12

## Abstract

The emergence of severe acute respiratory syndrome coronavirus 2 (SARS-CoV-2) has triggered a global pandemic, which severely endangers public health. Our and others’ works have shown that the angiotensin-converting enzyme 2 (ACE2)-containing exosomes (ACE2-exos) have superior antiviral efficacies, especially in response to emerging variants. However, the mechanisms of how the virus counteracts the host and regulates ACE2-exos remain unclear. Here, we identified that SARS-CoV-2 nonstructural protein 6 (NSP6) inhibits the production of ACE2-exos by affecting the protein level of ACE2 as well as tetraspanin-CD63 which is a key factor for exosome biogenesis. We further found that the protein stability of CD63 and ACE2 is maintained by the deubiquitination of proteasome 26S subunit, non-ATPase 12 (PSMD12). NSP6 interacts with PSMD12 and counteracts its function, consequently promoting the degradation of CD63 and ACE2. As a result, NSP6 diminishes the antiviral efficacy of ACE2-exos and facilitates the virus to infect healthy bystander cells. Overall, our study provides a valuable target for the discovery of promising drugs for the treatment of coronavirus disease 2019.

**IMPORTANCE:**

The outbreak of coronavirus disease 2019 (COVID-19) severely endangers global public health. The efficacy of vaccines and antibodies declined with the rapid emergence of severe acute respiratory syndrome coronavirus 2 (SARS-CoV-2) mutants. Angiotensin-converting enzyme 2-containing exosomes (ACE2-exos) therapy exhibits a broad neutralizing activity, which could be used against various viral mutations. Our study here revealed that SARS-CoV-2 nonstructural protein 6 inhibited the production of ACE2-exos, thereby promoting viral infection to the adjacent bystander cells. The identification of a new target for blocking SARS-CoV-2 depends on fully understanding the virus-host interaction networks. Our study sheds light on the mechanism by which the virus resists the host exosome defenses, which would facilitate the study and design of ACE2-exos-based therapeutics for COVID-19.

## INTRODUCTION

The global outbreak of coronavirus disease 2019 (COVID-19) caused by severe acute respiratory syndrome coronavirus 2 (SARS-CoV-2) poses a serious threat to public health and national economies due to its high pathogenicity and transmissibility (1, 2). SARS-CoV-2 is an enveloped β-coronavirus of the coronavirus family (3). It has a large, single-stranded positive-sense RNA genome of approximately 30 kb and consists of ORF1a, ORF1b, spike (S), envelope (E), membrane (M), nucleocapsid (N), ORF3a, ORF3b, ORF6, ORF7a, ORF8, ORF9b, and ORF10. The structures and functions of key virus-encoded proteins have been explored. The four structural proteins (S, E, M, and N) drive the assembly of virus particles (4). The accessory proteins (ORF3a, ORF3b, ORF6, ORF7a, ORF7b, ORF8, ORF9b, and ORF10) are thought to modulate the host response to facilitate viral infection (5–8).

The nonstructural proteins (NSP1–16) encoded by ORF1a and ORF1b perform unique functions that interfere with host homeostasis and make cellular conditions favorable for viral infection (3, 9–13). NSP1 is a leader protein that acts as a host translation inhibitor by binding to the 40S ribosomal subunit (14). NSP3 is a multi-domain protein that includes the papain-like protease (PLpro) domain involved in polyprotein processing. NSP3 coalesces with NSP4 to generate the double-membrane vesicles (DMVs), which are sites of viral replication (13, 15). NSP4 and ORF9b induce proinflammatory mitochondrial DNA release in inner membrane-derived vesicles (16). NSP5, also known as 3C-like protease (3CLpro), activates the nuclear factor-kappa Binding (NF-κB) pathway by upregulating the SUMOylation of MAVS (17, 18). NSP8 and NSP9 bind to the 7SL RNA in the signal recognition particle and interfere with protein trafficking to the cell membrane upon infection (19). Exosomes containing NSP12 or NSP13 can activate NF-κB and induce the production of inflammatory cytokines (20). NSP14 targets the type I interferon (IFN-I) receptor for lysosomal degradation (21). NSP15 inhibits the production of autophagosomes (21). Although the function of viral proteins has been widely studied, further understanding of virus-host interactions and molecular mechanisms remains critical for developing countermeasures against COVID-19.

NSP6 is a multi-transmembrane protein predicted to contain six to eight transmembrane domains (22). Several studies have identified that NSP6 plays pivotal roles in the folding, assembly, and replication of viral proteins (23–25). NSP6 has also been shown to engage in the formation of DMVs (26). The recent study also confirmed that NSP6 connects these DMVs with the endoplasmic reticulum (ER) (13). Mutations of a three-amino acid deletion (LSG, positions 105–107) and a unique amino substitution (I189V) located within NSP6 are associated with viral transmissibility and pathogenicity (23, 27). The polyubiquitination of NSP6 activates the NF-κB pathway and promotes the induction of proinflammatory responses (28). NSP6 can also trigger NLRP3-dependent caspase-1 activation, IL-1β/18 maturation, and pyroptosis of lung epithelial cells by impeding the acidification of lysosomes (25). Previous studies also showed that NSP6 induces smaller autophagosomes, which make the degradation of viral components less efficient (29–32). Another study showed that NSP6 inhibits IFN-I production by binding to tank-binding kinase 1 and blocks IFN-I signaling by preventing the phosphorylation of signal transducer and activator of transcription proteins 1 and 2 (6).

Extracellular vesicles (EVs) are mainly divided into endosome-origin “exosomes” with a diameter of 30–150 nm and plasma membrane-derived “microvesicles” with a diameter of 50–1,000 nm (33–35). Since their endocytic origin, exosomes are usually rich in endosome-associated proteins such as Rab GTPases, tetraspanins, and proteins of the endosomal sorting complex required for transport (36–38). The biogenesis of exosomes originates from the endocytic pathway, which includes several stages: endocytosis, early endosomes (EEs), late endosomes (LEs), multivesicular bodies (MVBs) formation, and exosome secretion. Ras-related protein Rab5 is enriched on EE. With the assistance of the Golgi complex, EEs mature into LEs, and Rab7 is enriched in LE. Vesicles can be retrogradely transported into the trans-Golgi network (TGN) at any time for recycling to endosomes or secretion. Rab9 is enriched in TGN. LEs eventually generate MVBs containing several intraluminal vesicles (ILVs). ILVs are the future exosomes. Rab27A and Rab27B mainly control the fusion of MVBs with cellular membranes, which is important for the release of exosomes. Tetraspanins, including CD9, CD63, and CD81, can induce membrane bending, which enables vesicle formation. These proteins are conserved transmembrane proteins enriched in exosomes (39). Given the excellent biocompatibility and high stability, exosomes have been widely applied in functional substances delivery against tumors and viral infection (40–43). Research proved that exosome-based strategies may be effective therapeutic tools for treating COVID-19 (44, 45).

SARS-CoV-2 mainly utilizes S protein to bind to human angiotensin-converting enzyme 2 (ACE2) on the cell surface to infect cells (46–48). In addition to developing neutralizing antibodies targeting spike protein, blocking the binding of S to ACE2 is considered an effective therapeutic strategy. ACE2 is widely distributed in epithelial cells of the human airway and intestine that are susceptible to SARS-CoV-2 infection. Besides, ACE2 can be secreted to the extracellular environment to competitively bind with S proteins and block the cell entry of SARS-CoV-2 (49). Clinical data showed that infected cells can produce EVs containing ACE2, which can attach to viral particles, thereby preventing virions from infecting healthy cells. It has been reported that upon SARS-CoV-2 infection, circulating ACE2-expressing EVs are increased and show potency to block SARS-CoV-2 virions (49). Defensosomes are a subset of exosomes that mediate protection against bacterial pore-forming toxins and are mobilized during bacterial infection in an autophagy protein-dependent manner (50). ACE2-containing defensosomes are also reported to be produced and serve as decoys to neutralize virions in response to SARS-CoV-2 infection (51). COVID-19 patients with high amounts of ACE2-positive exosomes in their bronchioalveolar lavage fluids (BALFs) were hospitalized for a shorter duration than patients with low amounts of ACE2-positive exosomes (52). Various studies have shown that engineered EVs expressing ACE2 can protect the host against SARS-CoV-2 infection, which further confirms the superior antiviral efficacy of ACE2-containing exosomes (ACE2-exos) (53, 54).

The expression and regulation of ACE2 are important for viral infection. Although current studies on ACE2-exos are continuing, our understanding of the delivery and dynamics of SARS-CoV-2 receptor ACE2 remains elusive. A previous study has shown that ACE2-exos were upregulated by IFN-α/β as a defense mechanism to inhibit SARS-CoV-2 infection (55). However, whether SARS-CoV-2 could impede the generation of ACE2-exos to counteract the host defenses is still unknown. In general, as the host defenses upgrade, the virus will evolve a series of countermeasures to evade the host response and facilitate its invasion and virulence (10).

Here, we screened individual SARS-CoV-2 proteins to evaluate their effects on ACE2-exos. Our data showed that NSP6 was greatly involved in the biogenesis of exosomes. The number of exosomes and level of functional ACE2 on exosomes significantly decreased by viral protein NSP6. We found that NSP6 hijacked the biogenesis of exosomes by interacting with CD63, which is critical for exosome formation. We also proved that NSP6 was able to suppress the antiviral function of ACE2-exos to promote viral invasion, which was reversed by the overexpression of CD63. Knockout of CD63 or overexpression of NSP6 showed the same inhibitory effect on ACE2-exos. Furthermore, we identified that proteasome 26S subunit, non-ATPase 12 (PSMD12), a component of 26S proteasome, participated in the maintenance of the stability of CD63 and ACE2 and protected CD63 and ACE2 from ubiquitin-mediated degradation. NSP6 bound PSMD12 to promote the degradation of CD63 and ACE2, reduce the production of exosomes and ACE2-exos, and ultimately facilitate viral infection to adjacent cells. The regulation of key proteins involved in exosome formation by NSP6 mainly occurred in EEs, LEs, and MVBs rather than during the initiation or release phase of exosome biogenesis. Our study deciphered the mechanism of the virus’s resistance to cellular exosome defenses and further clarified the interaction between virus and host, which will provide new therapeutic interventions for the treatment of COVID-19.

## RESULTS

### NSP6 inhibited the biogenesis of ACE2-exos

**Fig 1:**
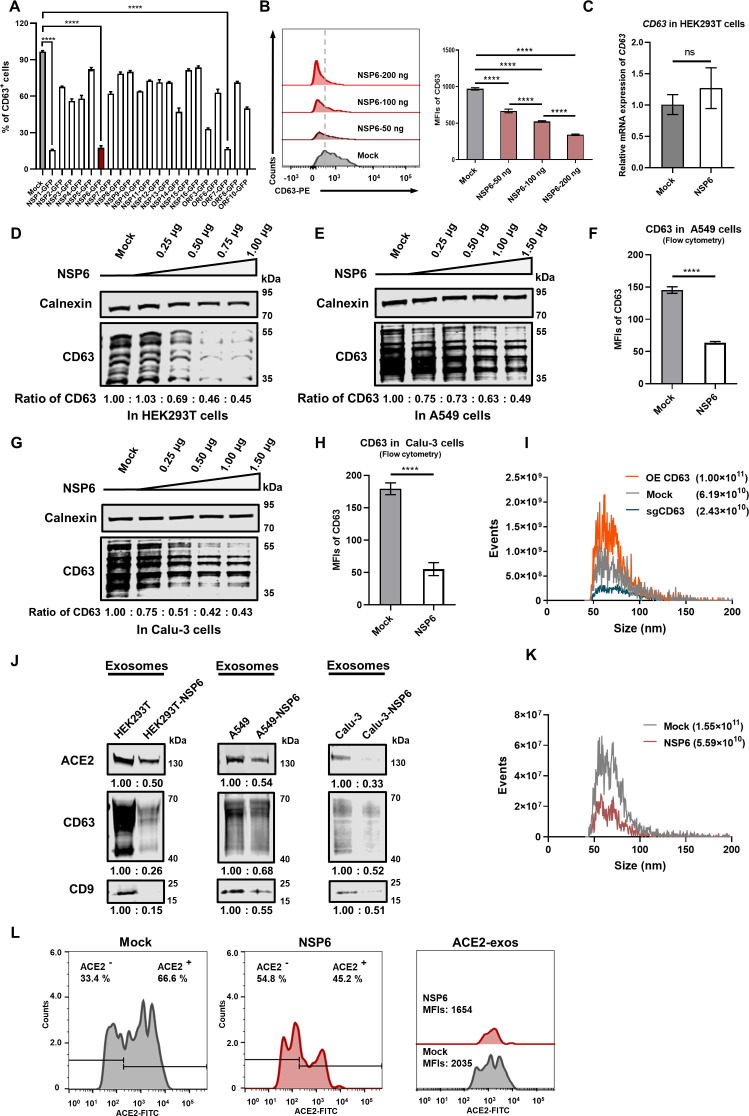
NSP6 inhibited the biogenesis of ACE2-exos. (**A**) The screening of viral proteins related to exosome formation. HEK293T cells were transfected with 300 ng of control empty vector or GFP-tagged SARS-CoV-2 protein-expressing plasmids, respectively. Cells were collected at 48 h post transfection (hpt). The frequencies of CD63-positive (CD63^+^) cells were analyzed by flow cytometry, which was first gated on GFP^+^ cells. (**B**) A dose-dependent manner of NSP6-mediated CD63 downregulation. HEK293T cells were transfected with various amounts (50 ng, 100 ng, and 200 ng) of GFP-tagged NSP6 (NSP6-GFP) plasmids, and mean fluorescence intensities (MFIs) of CD63 were analyzed by flow cytometry, gating on GFP^+^ cells. (**C**) The mRNA level of *CD63* after the overexpression of NSP6 in HEK293T cells, normalized to *GAPDH* mRNA expression. (**D**) The downregulation of CD63 by NSP6 was validated by Western blot in HEK293T cells. HEK293T cells were transfected with various amounts (0.25 µg, 0.50 µg, 0.75 µg, and 1.00 µg) of NSP6-GFP plasmids, followed by Western blot against calnexin (internal control) and CD63. (**E**) The downregulation of CD63 by NSP6 was validated by Western blot in A549 cells. A549 cells were transfected with various amounts (0.25 µg, 0.50 µg, 1.00 µg, and 1.50 µg) of NSP6-GFP plasmids, followed by Western blot against calnexin (internal control) and CD63. (**F**) The downregulation of CD63 by NSP6 was validated by flow cytometry in A549 cells. A549 cells were transfected with control empty vector or NSP6-GFP plasmids, and MFIs of CD63 were analyzed. (**G**) The downregulation of CD63 by NSP6 was validated by Western blot in Calu-3 cells. Calu-3 cells were transfected with various amounts (0.25 µg, 0.50 µg, 1.00 µg, and 1.50 µg) of NSP6-GFP plasmids, followed by Western blot against calnexin (internal control) and CD63. (**H**) The downregulation of CD63 by NSP6 was validated by flow cytometry in Calu-3 cells. Calu-3 cells were transfected with control empty vector or NSP6-GFP plasmids, and MFIs of CD63 were analyzed. (**I**) The size and concentration of purified exosomes, which were derived from CD63-overexpressing (OE CD63) or CD63-knockout (sgCD63) HEK293T cells (OE CD63 and sgCD63), were detected by Nano-FCM. (**J**) Western blot analysis of purified exosomes derived from HEK293T, A549, and Calu-3 cells which were co-overexpressed with NSP6 (HEK293T-NSP6, A549-NSP6, and Calu-3-NSP6). (**K**) The size and concentration of purified exosomes, which were derived from NSP6-overexpressing HEK293T cells or wild-type HEK293T cells, were detected by Nano-FCM. (**L**) Effect of NSP6 on proportions and MFIs of ACE2-exos. The ratio of ACE2-positive (ACE2^+^) and ACE2-negative (ACE2^−^) exosomes derived from wild-type HEK293T cells (Mock) or NSP6-overexpressing HEK293T cells (NSP6) and the MFIs of ACE2^+^ exosomes were analyzed by Nano-FCM. The data were shown as mean ± SD (error bars) in triplicate. *P*-values were calculated by one-way analysis of variance (ANOVA) tests (**A and B**) or Student’s *t*-test (**C, F,** and **H**). ****P < 0.0001; ns, not significant.

To elucidate whether the viral proteins can regulate ACE2-exos to resist the host defenses, we cloned major genes of SARS-CoV-2 into a mammalian expression plasmid with a GFP tag and confirmed the expression of these GFP-tagged proteins by Western blot (Fig. S1A). We transfected HEK293T cells with these plasmids expressing individual SARS-CoV-2 proteins, including 16 nonstructural proteins and five accessory proteins. An empty vector plasmid was transfected as the negative control. The classical exosomal biomarkers, including CD9, CD81, and CD63, were used as the exosome indicators to screen SARS-CoV-2 proteins, which might be involved in the biogenesis of exosomes. At 24 h post transfection, cells were harvested for flow cytometry analysis. We found that NSP1, NSP6, and ORF7 showed excellent inhibitory effects on the expression of CD63 (Fig. 1A). Given that it has been reported that NSP1 inhibits the global translation by sterically occluding the entrance region of the mRNA channel in ribosomal complexes, NSP1-mediated CD63 downregulation seemed to be a logical phenomenon (11). NSP6 and ORF7 also significantly reduced the frequency of CD63^+^ cells. Since several studies have confirmed that NSP6 affects pathogenicity and transmissibility and participates in the formation of DMVs, NSP6 is most likely related to endosome circulation (13, 23). Besides, CD9 and CD81 were more significantly inhibited by NSP6 rather than ORF7 (Fig. S1B through E). Thus, NSP6 merited being further studied.

Both the frequencies and mean fluorescence intensities (MFIs) of CD63^+^ cells were significantly downregulated upon NSP6 overexpression (Fig. 1A and B). NSP6 induced the downregulation of MFIs of both CD63 and CD81 in dose-dependent manners (Fig. 1B; Fig. S1C). We also identified that NSP6 did not affect the transcriptional levels of *CD63* mRNAs in HEK293T cells (Fig. 1C). Western blot analysis also revealed that NSP6 induced the downregulation of CD63 in a dose-dependent manner in HEK293T cells (Fig. 1D). Furthermore, the downregulation of CD63 mediated by NSP6 was also verified in lung epithelial cell lines A549 and Calu-3 by Western blot and flow cytometry analysis (Fig. 1E through H).

Next, we validated the effect of CD63 on the secretion of exosomes. We constructed HEK293T-CD63-GFP (OE CD63) and HEK293T-sgCD63 (sgCD63) cell lines by overexpression or knockout of CD63, respectively. The knockout efficiency of CD63 was verified by flow cytometry, and the sg-CD63-3 construct was selected for subsequent studies (Fig. S1F). The exosomes were purified from cell supernatant by differential ultracentrifugation and verified for successful purification by transmission electron microscopy (TEM) to characterize the morphology and nano-flow cytometry (Nano-FCM) to evaluate the sizes (Fig. S1G; Fig. 1I). By Nano-FCM analysis, we found that the deletion of CD63 reduced the number of exosomes, while overexpression of CD63 increased the number of exosomes (Fig. 1I). These results suggested that CD63 positively regulated the secretion of exosomes. Western blot analysis also revealed that the deletion of CD63 reduced the number of ACE2-exos, while overexpression of CD63 increased the number of ACE2-exos (Fig. S1H).

Given that NSP6 showed suppressive effects on the expression of CD63, we speculated that NSP6 might affect total exosomes as well as ACE2-exos. We constructed the cell lines HEK293T-NSP6, A549-NSP6, and Calu-3-NSP6 by overexpressing NSP6 proteins within these cells. Western blot analysis revealed that ACE2-exos were significantly inhibited by NSP6 within all three NSP6-overexpressing cell lines (Fig. 1J). Nano-FCM analysis showed that the numbers of total exosomes were reduced upon NSP6 overexpression (Fig. 1K). The proportion of ACE2-exos and the MFIs of ACE2-exos also decreased when NSP6 was overexpressed (Fig. 1L). Immune-electron microscopy (iEM) images showed that ACE2 proteins existed on the surfaces of exosomes (Fig. S1I). Collectively, these results showed that NSP6 inhibited the production of exosomes and impeded the secretion of ACE2 by affecting the biogenesis of exosomes.

### NSP6 antagonized the blocking effect of ACE2-exos and promoted viral infection

Previously, our group confirmed that ACE2-exos were able to inhibit SARS-CoV-2 replication by competitively blocking the virus entry, the scheme of which was shown in Fig. S2A (55). Thus, we first evaluated the blocking effect of ACE2-exos on pseudotyped SARS-CoV-2 virus infection. The exosomes that were derived from ACE2-overexpressing HEK293 cells (ACE2-exos group) enhanced the blocking effect and inhibited viral infection compared with exosomes derived from wild-type HEK293T cells (mock group) (Fig. S2B). We further confirmed that ACE2-exos derived from HEK293T-hACE2 cells blocked cell entry of multiple pseudotyped SARS-CoV-2 variants including B.1.1.7 (alpha), B.1.351 (beta), B.1.617.1 (kappa), C.37 (lambda), BA.1 (omicron subvariant), and BA.2 (omicron subvariant), supporting the broad-spectrum antiviral mechanism of ACE2-exos for therapeutics development (Fig. S2C).

**Fig 2:**
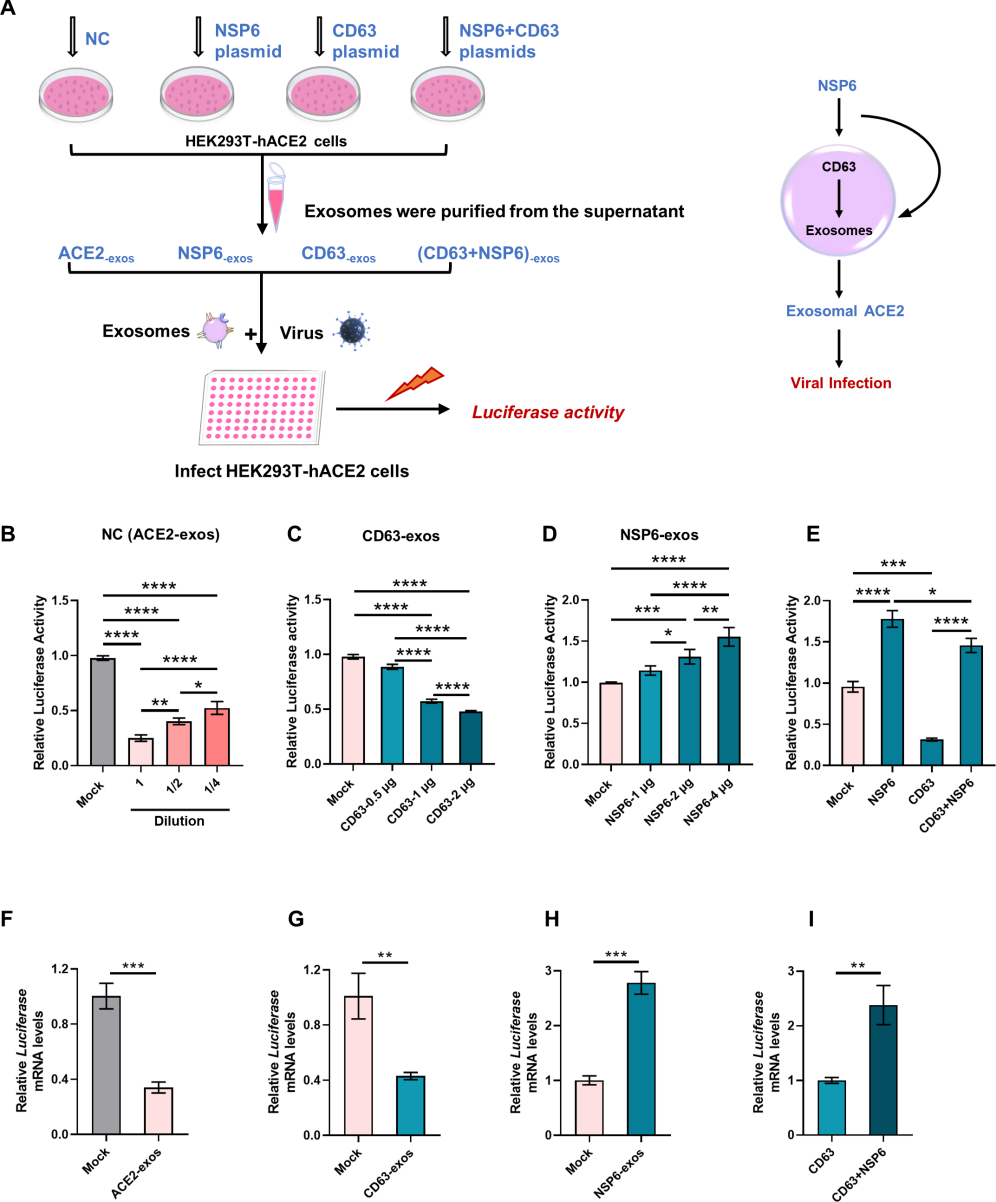
NSP6 antagonized the blocking effect of ACE2-exos and promoted viral infection. (**A**) Schematic of SARS-CoV-2 pseudotyped virus infection experiment. HEK293T-hACE2 cells were transfected with the plasmids encoding NSP6 or CD63 or co-transfected with CD63 and NSP6. The empty vector was transfected as negative control (NC). Supernatants of each group were collected and purified for exosomes at 24 hpt. Pseudotyped SARS-CoV-2 viruses were mixed with purified exosomes for 15 min at room temperature, followed by adding into HEK293T-hACE2 or A549-hACE2 cells seeded into 96-well plates. Cells were harvested to detect relative luciferase activity and *luciferase* mRNA levels, which represented viral infectivity at 24 h post incubation. (**B**) ACE2-exos blocked viral infection in HEK293T-hACE2 cells in a dose-dependent manner. Pseudotyped SARS-CoV-2 viruses were mixed with twofold serially diluted (1, 1/2, and 1/4) ACE2-exos for 15 min at room temperature, followed by infecting HEK293T-hACE2 cells. The relative luciferase activities within each group were measured at 24 h post infection (hpi). (**C**) CD63 enhanced the blocking effect of ACE2-exos on viral infection in a dose-dependent manner. HEK293T-hACE2 cells were transfected with various amounts (0.50 µg, 1.00 µg, and 2.00 µg) of CD63 plasmids. Pseudotyped SARS-CoV-2 viruses were co-incubated with CD63-overexpressing ACE2-exos (CD63-exos), followed by infecting HEK293T-hACE2 cells. The relative luciferase activities within each group were analyzed at 24 hpi. (**D**) NSP6 inhibited the blocking effect of ACE2-exos on viral infection in a dose-dependent manner. HEK293T-hACE2 cells were transfected with various amounts (1.00 µg, 2.00 µg, and 4.00 µg) of plasmids expressing NSP6. Pseudotyped SARS-CoV-2 viruses were co-incubated with NSP6-overexpressing ACE2-exos (NSP6-exos), followed by infecting HEK293T-hACE2 cells. The relative luciferase activities within each group were analyzed at 24 hpi. (**E**) The effect of ACE2-exos derived from HEK293T-hACE2 cells overexpressed with NSP6 and CD63 or co-overexpressed with CD63 and NSP6 on viral infection within HEK293T-hACE2 cells. Pseudotyped SARS-CoV-2 viruses were co-incubated with NSP6-overexpressing ACE2-exos (NSP6), CD63-overexpressing ACE2-exos (CD63), or CD63 and NSP6 co-overexpressing ACE2-exos (CD63 + NSP6), followed by infecting HEK293T-hACE2 cells. The relative luciferase activities within each group were analyzed at 24 hpi. (**F**) Real-time quantitative PCR analysis of the mRNA level of *luciferase* in HEK293T-hACE2 cells, normalized to *GAPDH* mRNA expression. Pseudotyped SARS-CoV-2 viruses were co-incubated with or without ACE2-exos, followed by infecting HEK293T-hACE2 cells. The mRNA levels of *luciferase* within each group were measured at 24 hpi. (**G-H**) The mRNA level of *luciferase* in HEK293T-hACE2 cells normalized to *GAPDH* mRNA expression. Pseudotyped SARS-CoV-2 viruses were co-incubated with CD63-overexpressing ACE2-exos (CD63) (**G**), NSP6-overexpressing ACE2-exos (NSP6) (**H**), or CD63 and NSP6 co-overexpressing ACE2-exos (CD63 + NSP6) (**I**), followed by infecting HEK293T-hACE2 cells. The mRNA levels of *luciferase* within each group were measured at 24 hpi. The data were shown as mean ± SD (error bars) in triplicate. *P*-values were calculated by one-way ANOVA tests (**B–E**) or Student’s *t*-test (**F–I**). *P < 0.05, **P < 0.01, ***P < 0.001, ****P < 0.0001.

In order to investigate whether NSP6 could antagonize the blocking effect exerted by ACE2-exos and promote viral infection, we performed pseudotyped virus infection experiments (Fig. 2A). Serially diluted exosomes derived from HEK293T-hACE2 cells significantly inhibited the viral infection in dose-dependent manners (Fig. 2B). Compared with HEK293T-hACE2 cell-derived exosomes, exosomes derived from HEK293T-hACE2 cells co-overexpressing CD63 exhibited more efficiencies on blocking viral infection in dose-dependent manners (Fig. 2C), while the exosomes derived from HEK293T-hACE2 cells which were co-overexpressed with NSP6 weakened the blocking effect of ACE2-exos in dose-dependent manners, which in turn promoted viral infection (Fig. 2D). Finally, we identified that the blocking effect from CD63 overexpression could be antagonized by co-overexpressing NSP6 to restore viral infection (Fig. 2E). Moreover, the mRNA levels of *luciferase* gene were also evaluated (56). Consistent with the results of luciferase activities, ACE2-exos downregulated the mRNA levels of *luciferase* genes in pseudotyped SARS-CoV-2 virus-infected HEK293T-hACE2 cells (Fig. 2F). Compared with ACE2-exos, exosomes derived from HEK293T-hACE2 cells which were co-overexpressed with CD63 further downregulated the mRNA levels of *luciferase* genes (Fig. 2G). In contrast, exosomes derived from HEK293T-hACE2 cells, which were co-overexpressed with NSP6, upregulated the mRNA levels of *luciferase* genes (Fig. 2H). We further found that the downregulation of *luciferase* mRNA by CD63 could be antagonized by overexpressing NSP6 to increase the mRNA level of *luciferase* (Fig. 2I).

NSP6 also acted in the same way for CD9 and CD81. Compared with the exosomes derived from HEK293T-hACE2 cells, which were co-overexpressed with CD9 or CD81, the exosomes derived from HEK293T-hACE2 cells, which were co-overexpressed NSP6 with CD9 or CD81 reduced the blocking effect and promoted viral infection (Fig. S2D and E). We also confirmed that ACE2, CD63, and NSP6 played similar roles upon infecting A549-hACE2 cells. Compared with HEK293T-hACE2 cell-derived exosomes, exosomes derived from HEK293T-hACE2 cells, which were co-overexpressed with NSP6, reduced the blocking effect of ACE2-exos and promoted viral infection to A549-hACE2 cells (Fig. S2F). Exosomes derived from HEK293T-hACE2 cells, which were co-overexpressed with CD63, enhanced the blocking effect and inhibited viral infection to A549-hACE2 cells, and the blocking effect from CD63 overexpression could be decreased by co-overexpressing NSP6 to restore viral infection (Fig. S2F). Our above results indicated that NSP6 could antagonize the blocking effect of ACE2-exos, which eventually promoted viral infection.

### NSP6 hijacked the CD63-mediated formation of ACE2-exos in the endocytic pathway

**Fig 3:**
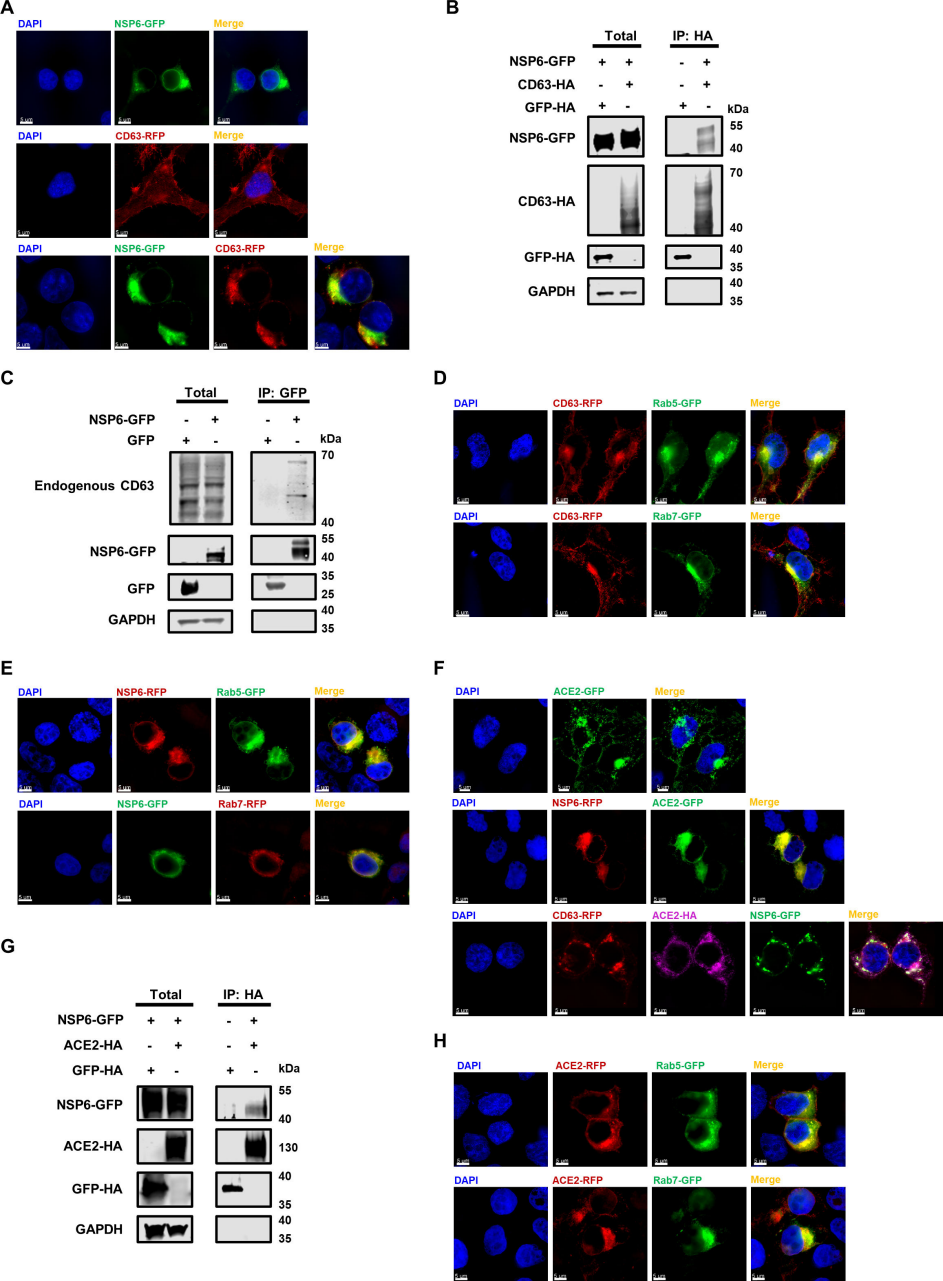
NSP6 hijacked the CD63-mediated formation of ACE2-exos in the endocytic pathway. (**A**) The localization of NSP6 and CD63 within HEK293T cells. Cells were transfected with NSP6-GFP or CD63-RFP or co-transfected with NSP6-GFP and CD63-RFP. Cells were harvested for conducting immunofluorescence (IF) assay at 48 hpt (**B**) The interaction between NSP6 and CD63 in HEK293T cells. Cells were transfected with NSP6-GFP along with GFP-HA or CD63-HA, respectively. Cells were lysed and immunoprecipitated with anti-HA beads at 48 hpt. The expressions of NSP6-GFP, CD63-HA, GFP-HA, and GAPDH within both total and IP samples were subjected to Western blot assays. (**C**) The interaction between NSP6 and endogenous CD63 in HEK293T cells. Cells were transfected with NSP6-GFP or GFP. Cells were lysed and immunoprecipitated with anti-GFP beads at 48 hpt. The expressions of endogenous CD63, NSP6-GFP, GFP, and GAPDH within both total and IP samples were subjected to Western blot assays. (**D**) The co-localization of CD63 with Rab5 and Rab7 in HEK293T cells. Cells were transfected with CD63-RFP along with Rab5-GFP and Rab7-GFP, respectively. Cells were subjected to IF assay at 48 hpt. (**E**) The co-localization of NSP6 with Rab5 and Rab7 in HEK293T cells. Cells were co-transfected with NSP6-RFP and Rab5-GFP or co-transfected with NSP6-GFP and Rab7-RFP, followed by IF assay at 48 hpt. (**F**) The localization of ACE2, NSP6, and CD63 in HEK293T cells. Cells were transfected with ACE2-GFP, co-transfected with NSP6-RFP and ACE2-GFP, or co-transfected with CD63-RFP, NSP6-GFP, and ACE2-HA. Samples were subjected to IF assay at 48 hpt. ACE2 proteins were stained with anti-HA antibodies. (**G**) The interaction between NSP6 and ACE2 in HEK293T cells. Cells were co-transfected with NSP6-GFP along with GFP-HA or ACE2-HA, respectively. At 48 hpt, cells were lysed and immunoprecipitated with anti-HA beads, followed by Western blot assay to indicate the expression of NSP6-GFP, ACE2-HA, GFP-HA, and GAPDH within both total and IP samples. (**H**) The co-localization of ACE2 with Rab5 and Rab7 in HEK293T cells. Cells were co-transfected with ACE2-RFP along with Rab5-GFP and Rab7-GFP, respectively, followed by IF assay at 48 hpt. The nucleus was stained with 4′,6-diamidino-2-phenylindole dihydrochloride (DAPI) (blue). Scale bars represented 5 µm. All samples were imaged to obtain at least three images.

To elucidate the mechanisms of how NSP6 affected ACE2-exos, we performed immunofluorescence (IF) assays to evaluate the localization of NSP6, CD63, and ACE2. CD63, as a tetraspanin protein, mainly distributes on the cell membrane and endosome membrane (37, 57). In contrast, NSP6 is a multi-transmembrane protein that localizes on the ER membrane and in the perinuclear space (24, 58). After co-transfecting GFP-tagged NSP6 (NSP6-GFP) with CD63-RFP, we found that most CD63 proteins were redistributed from the membrane surface into the cytoplasm and significantly co-localized with NSP6 proteins (Fig. 3A; Fig. S3A). We further performed immunoprecipitation (IP) assays to evaluate the interaction between NSP6 and CD63. The result showed that NSP6 is bound to both exogenous and endogenous CD63 (Fig. 3B and C). These results indicated that NSP6 might hijack CD63, which is critical for exosome biogenesis. To verify whether NSP6 interfered with the endocytic pathway, we co-transfected CD63 or NSP6 with Rab proteins involved in vesicle trafficking and membrane fusion (59, 60). We found that both CD63 and NSP6 strikingly co-localized with Rab5 (a biomarker of EEs), Rab7 (a biomarker of LEs and MVBs), and Rab9 (a biomarker of TGN), but not with Rab27A and Rab27B (Fig. 3D and E; Fig. S3B through I). These results suggested that NSP6 might regulate CD63 during early endosomes, late endosomes, and MVBs.

We also conducted an IF analysis to elucidate the relationship between ACE2 and NSP6. We found that ACE2 significantly co-localized with NSP6 (Fig. 3F; Fig. S4A). The interaction between NSP6 and ACE2 was confirmed by IP assays (Fig. 3G). ACE2 also co-localized with CD63 (Fig. S4B). However, CD63 and ACE2 proteins were redistributed into the cytoplasm after the co-overexpression of NSP6 (Fig. 3F; Fig. S4C). ACE2 also showed co-localization with Rab5 and Rab7 (Fig. 3H; Fig. S4D through E). Based on the above results, we speculated that CD63 might assist ACE2 secretion from the cytoplasm into the extracellular environment, which is restricted by NSP6 via interfering with the endocytic pathway.

### PSMD12 served as a key protein involved in the regulation of ACE2-exos

To clarify how SARS-CoV-2 downregulated the key exosomal protein CD63 and the secretion of ACE2 through NSP6, we performed mass spectrometry to find out which proteins might be involved in the underlying mechanisms. We overexpressed GFP or GFP-tagged NSP6 (NSP6-GFP) in HEK293T cells. Transfected cells were harvested, followed by the enrichment of GFP or NSP6-GFP proteins as well as corresponding co-immunoprecipitated proteins. We used SDS-PAGE to separate the enriched proteins and excised distinct protein bands, followed by in-gel digestion with trypsin. The digested peptides were identified by nanoscale liquid chromatography-mass spectrometry/mass spectrometry (LC-MS/MS) and annotated by PEAKS Studio (Fig. S5A and B).

**Fig 4:**
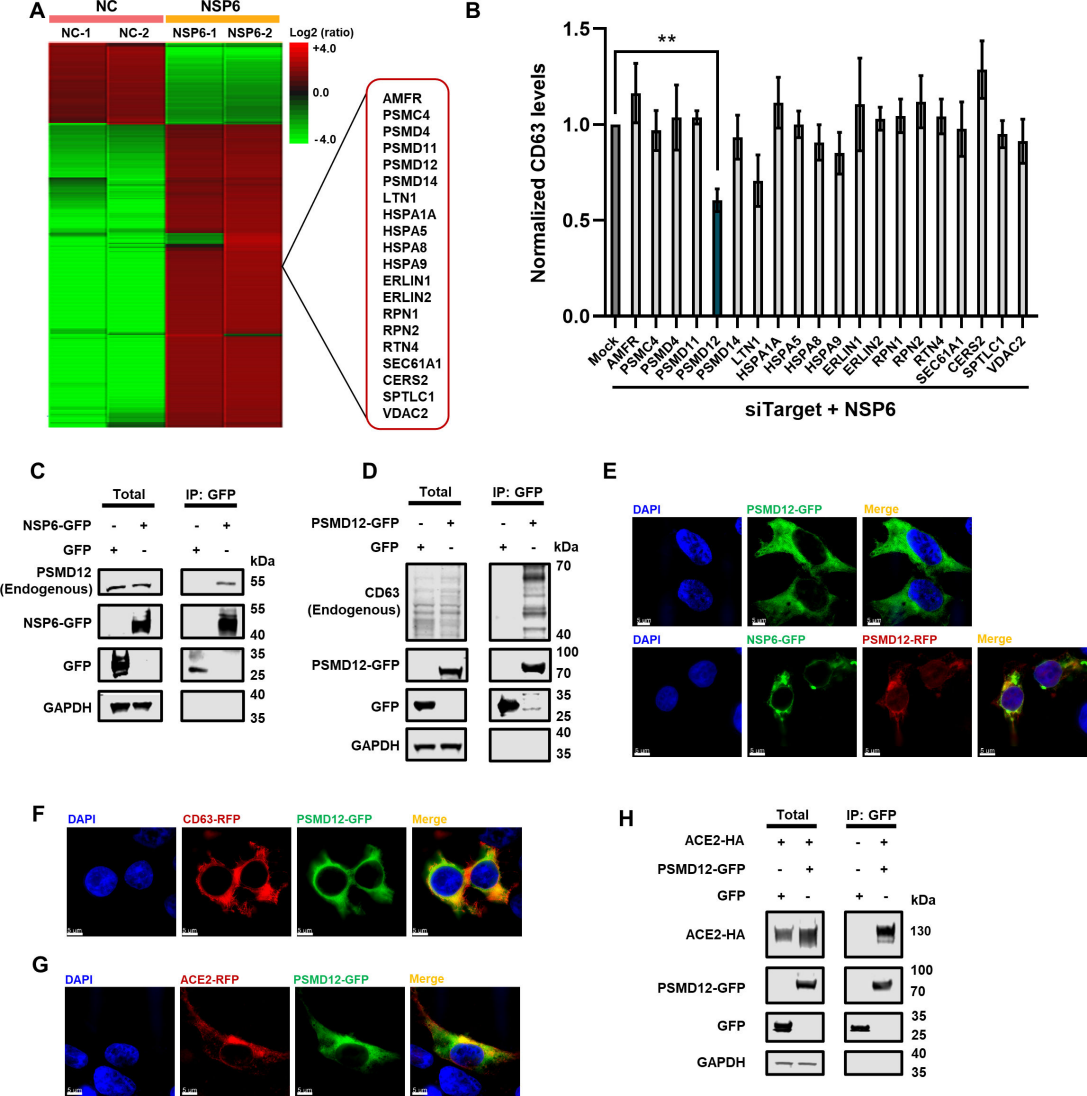
PSMD12 was involved in the regulation of ACE2-exos. (**A**) The heatmap of NSP6-enriched proteins. NSP6-GFP was overexpressed in HEK293T cells. Cells were lysed and immunoprecipitated with anti-GFP beads at 48 hpt. The negative control groups were transfected with an empty vector (NC). The IP samples were subjected to SDS-PAGE and developed with silver staining. The whole lane of each group was cut into several gel slices and proceeded to in-gel digestion and LC-MS/MS to identify NSP6-enriched proteins. (**B**) Screening of LC-MS/MS-identified proteins by siRNA library. HEK293T cells were transfected with siRNAs targeting each gene, followed by transfecting with NSP6-GFP-expressing plasmids. Cells were analyzed by flow cytometry at 48 hpt, gated on GFP^+^ cells. The assay was repeated three times. (**C**) The interaction between NSP6 and endogenous PSMD12 in HEK293T cells. Cells were transfected with NSP6-GFP or GFP. Cells were lysed and immunoprecipitated with anti-GFP beads at 48 hpt, followed by Western blot assay to indicate the expression of endogenous PSMD12, NSP6-GFP, GFP, and GAPDH within both total and IP samples. (**D**) The interaction between PSMD12 and endogenous CD63 in HEK293T cells. Cells were transfected with PSMD12-GFP or GFP. Cells were lysed and immunoprecipitated with anti-GFP beads at 48 hpt. The expressions of endogenous CD63, PSMD12-GFP, GFP, and GAPDH within both total and IP samples were subjected to Western blot assays. (**E**) The co-localization of PSMD12 and NSP6 in HEK293T cells. Cells were transfected with PSMD12-GFP or co-transfected with NSP6-GFP and PSMD12-RFP, followed by IF assay at 48 hpt. (**F-G**) The co-localization of PSMD12 with CD63 and ACE2 in HEK293T cells. Cells were co-transfected with PSMD12-GFP and CD63-RFP (**F**) or co-transfected with PSMD12-GFP and ACE2-RFP (**G**), followed by IF assay at 48 hpt. (**H**) The interaction between PSMD12 and ACE2 in HEK293T cells. Cells were transfected with ACE2-HA along with GFP or PSMD12-GFP, respectively. Cells were lysed and immunoprecipitated with anti-GFP beads at 48 hpt. The nucleus was stained with DAPI (blue). Scale bars represented 5 µm. All samples were imaged to obtain at least three images. The data were shown as mean ± SD (error bars) in triplicate. *P*-values were calculated by one-way ANOVA tests (**B**). **P < 0.01.

Many proteins significantly enriched by NSP6 were selected for further study (Fig. 4A). AMFR, PSMC4, PSMD4, PSMD11, PSMD12, PSMD14, and LTN1 were involved in proteasomal systems (61–68). HSPA1A, HSPA5, HSPA8, and HSPA9 were molecular chaperones implicated in a variety of cellular processes (61, 69–71). ERLIN1, ERLIN2, RPN1, RPN2, RTN4, and SEC61A1 were related to endoplasmic reticulum-associated degradation (61, 72–74). CERS2, SPTLC1, and VDAC2 may be involved in lipid metabolism and substance trafficking associated with vesicle production (75–78). To confirm which factors are involved in the regulation of NSP6 to CD63, we performed a screening with an siRNA library targeted to the above proteins. Interestingly, we found that the knockdown of PSMD12 promoted the downregulation of CD63 (Fig. 4B). PSMD12, as a component of 26S proteasome, plays a key role in the maintenance of protein homeostasis by regulating the ubiquitination of captured substrates (79). Co-immunoprecipitation (Co-IP) experiments revealed that both endogenous and exogenous PSMD12 interacted with NSP6 (Fig. 4C; Fig. S5C). The interaction between PSMD12 and CD63 was also confirmed by IP assays (Fig. 4D). We also found that the expression of CD63 was enhanced by PSMD12 (Fig. S5D). IF data also showed that PSMD12 co-localized with both NSP6 and CD63 (Fig. 4E and F; Fig. S5E and F). To investigate the influence of PSMD12 on ACE2, we first conducted IF and IP experiments to investigate the interaction of PSMD12 and ACE2. The results showed that PSMD12 co-localized with ACE2 (Fig. 4G; Fig. S5G). Besides, PSMD12 is also co-immunoprecipitated with ACE2 (Fig. 4H).

**Fig 5:**
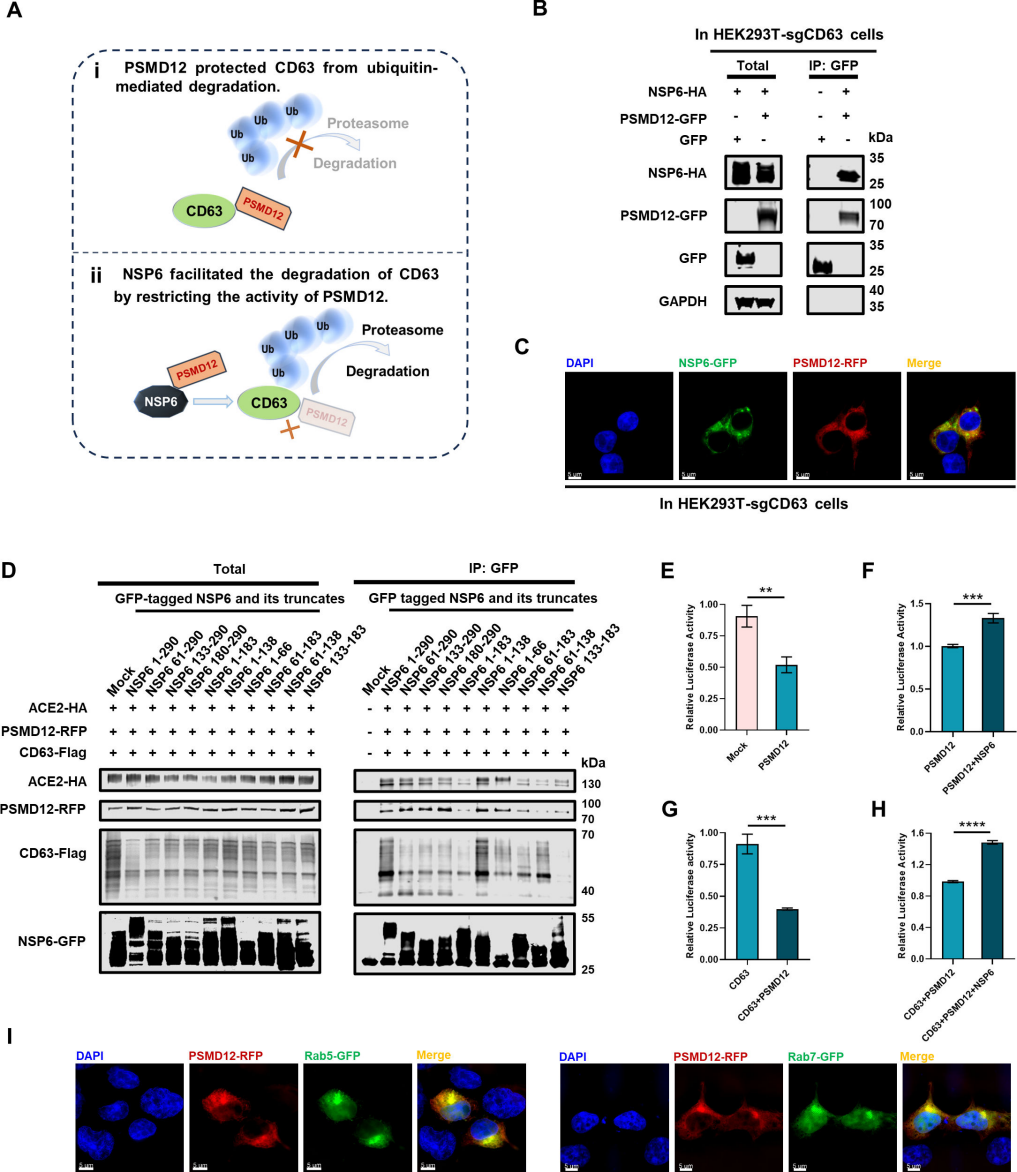
PSMD12 participated in the regulation of ACE2-exos by NSP6. (**A**) Schematic of PSMD12 involved in NSP6-induced regulation of CD63. (i) PSMD12 protected CD63 from ubiquitin-mediated degradation. (ii) NSP6 promoted the degradation of CD63 by binding to PSMD12 to restrict its deubiquitination activities. (**B**) The interaction between PSMD12 and NSP6 in HEK293T-sgCD63 cells. Cells were transfected with NSP6-HA along with GFP or PSMD12-GFP, respectively. At 48 h post transfection, cells were lysed and immunoprecipitated with anti-GFP beads. (**C**) The co-localization between NSP6 and PSMD12 in HEK293T-sgCD63 cells. Cells were transfected with NSP6-GFP along with PSMD12-RFP. (**D**) The interaction between NSP6 and its mutants (NSP6 61–290, NSP6 133–290, NSP6 180–290, NSP6 1–183, NSP6 1–138, NSP6 1–66, NSP6 61–183, NSP6 61–138, and NSP6 133–183) with CD63, ACE2, and PSMD12 in HEK293T cells. Cells were co-transfected with empty vector (GFP) or GFP-tagged NSP6 and corresponding truncates, along with CD63-Flag, ACE2-HA, and PSMD12-RFP. Cells were lysed and immunoprecipitated with anti-GFP beads at 48 hpt. (**E**) The effect of ACE2-exos derived from HEK293T-hACE2 cells overexpressing with PSMD12 on viral infection within HEK293T-hACE2 cells. HEK293T-hACE2 cells were overexpressed with empty vector (mock group) and PSMD12-encoding plasmid (PSMD12 group), respectively, and the supernatants were collected and purified for exosomes at 24 hpt. Pseudotyped SARS-CoV-2 viruses were mixed with purified exosomes for 15 min at room temperature, followed by adding into HEK293T-hACE2 cells seeded into 96-well plates. The relative luciferase activities were measured to indicate the infectivity. (**F–H**) The effect of ACE2-exos derived from HEK293T-hACE2 cells overexpressing with PSMD12, PSMD12 and NSP6, CD63, PSMD12 and CD63, and NSP6 with PSMD12 and CD63 on viral infection within HEK293T-hACE2 cells. HEK293T-hACE2 cells were transfected with PSMD12-encoding plasmids or co-transfected with PSMD12 and NSP6-encoding plasmids (**F**), transfected with CD63-encoding plasmids, or co-transfected with CD63 and PSMD12-encoding plasmids (**G**), co-transfected with CD63 and PSMD12-encoding plasmids, or co-transfected with CD63, PSMD12, and NSP6-encoding plasmids (**H**). The supernatants were collected and purified for exosomes at 24 hpt. Pseudotyped SARS-CoV-2 viruses were co-incubated with PSMD12-overexpressing ACE2-exos (PSMD12 group); PSMD12 and NSP6 co-overexpressing ACE2-exos (PSMD12 + NSP6 group); CD63-overexpressing ACE2-exos (CD63 group); CD63 and PSMD12 co-overexpressing ACE2-exos (CD63 + PSMD12 group); or CD63, PSMD12, and NSP6 co-overexpressing ACE2-exos (CD63 + PSMD12 + NSP6 group) respectively, followed by infecting HEK293T-hACE2 cells. The relative luciferase activities within each group were analyzed at 24 hpi. (**I**) The co-localization of PSMD12 with Rab5 and Rab7 in HEK293T cells. Cells were transfected with PSMD12-RFP along with Rab5-GFP and Rab7-GFP, respectively. At 24 hpt, the distributions of these proteins were visualized. The nucleus was stained with DAPI (blue). Scale bars represented 5 µm. All samples were imaged to obtain at least three images. The data were shown as mean ± SD (error bars) in triplicate. *P*-values were calculated using the Student’s *t*-test (**E-H**). **P < 0.01, ***P < 0.001, ****P < 0.0001.

We speculated that PSMD12 might be involved in maintaining the stability of CD63, while the binding of NSP6 to PSMD12 might inhibit its stabilization activity (Fig. 5A). In order to determine the role of PSMD12 in the downregulation of CD63 mediated by NSP6, the co-localization and interaction between PSMD12 and NSP6 was re-confirmed in the CD63-knockout cell lines named HEK293T-sgCD63 (Fig. 5B and C; Fig. S6A). To identify the crucial domain of NSP6 that interacted with CD63, ACE2, and PSMD12, we constructed 12 truncated mutants based on the transmembrane helix of full-length NSP6 (NSP6 1–290) predicted by TMHMM (80). We utilized a sequential deletion strategy to generate NSP6 truncates, including several variants with N- and/or C-terminal deletions or retention of both N- and C-terminals, such as NSP6 61–290, NSP6 133–290, NSP6 180–290, NSP6 1–183, NSP6 1–138, NSP6 1–66, NSP6 61–183, NSP6 61–138, NSP6 133–183, NSP6 Δ61–132, NSP6 Δ133–179, and NSP6 Δ180–232 with GFP tag (Fig. S6B). Co-IP analysis showed that the full-length NSP6 and NSP6 1–138 displayed a strong interaction with CD63 and ACE2, while NSP6 133–183 showed a minimal interaction with CD63. Furthermore, nearly all NSP6 truncates interacted with PSMD12 (Fig. 5D). These truncates, which retained the N- and C- terminals while deleting the middle segment of NSP6, also failed to abolish their interactions with CD63, ACE2, and PSMD12 (Fig. S6C). These findings indicated that multiple domains of the multi-transmembrane protein NSP6 collaborated to facilitate and sustain the interaction process.

The effect of PSMD12 on ACE2-exos-mediated inhibition of viral infection was also evaluated. Results showed that the exosomes derived from PSMD12-overexpressing HEK293T-hACE2 cells exhibited a more potent antiviral effect than those derived from HEK293T-hACE2 cells (Fig. 5E). The enhanced antiviral effect resulting from PSMD12 overexpression could be counteracted by co-overexpressing NSP6 to restore infection (Fig. 5F). The exosomes obtained from HEK293T-hACE2 cells co-overexpressing of CD63 and PSMD12 demonstrated a more robust antiviral effect than those obtained from HEK293T-hACE2 cells overexpressing CD63 only and further inhibited viral infection (Fig. 5G). The enhanced antiviral effect from CD63 and PSMD12 co-overexpression could be compromised upon NSP6 overexpression, thereby facilitating viral infection (Fig. 5H). Furthermore, PSMD12 also showed co-localization with Rab5 and Rab7, which was consistent with our speculation that PSMD12 was involved in the regulation of CD63 by NSP6 in the endocytic pathway (Fig. 5I; Fig. S6D and E). Our above results indicated that PSMD12 might be hijacked by NSP6 to downregulate CD63 and ACE2-exos.

### NSP6 promoted the ubiquitin-mediated degradation of CD63

**Fig 6:**
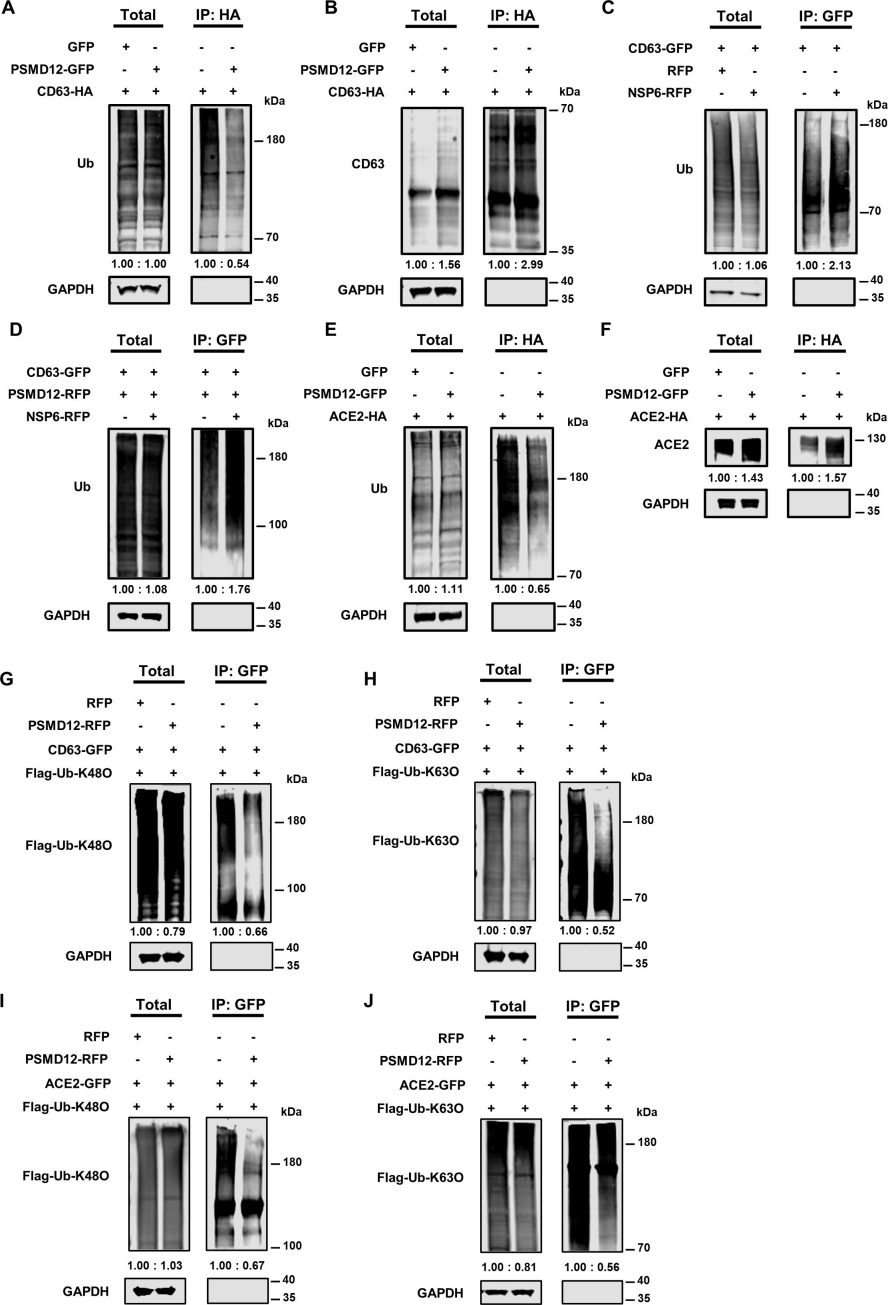
NSP6 promoted the ubiquitin-mediated degradation of CD63. (**A**) PSMD12 reduced the ubiquitination levels of CD63. HEK293T cells were co-transfected with CD63-HA along with GFP or PSMD12-GFP, respectively. At 48 hpt, 10 µM MG-132 was added into the culture medium and incubated for 12 h. Cells were lysed and immunoprecipitated with anti-HA beads. The expression levels of ubiquitin-conjugated proteins and GAPDH within both total and IP samples were evaluated by Western blot assays. (**B**) PSMD12 safeguarded the expression levels of CD63. HEK293T cells were treated as in **A**. The expression levels of CD63 and GAPDH within both total and IP samples were evaluated by Western blot assays. (**C**) NSP6 promoted the ubiquitination of CD63. HEK293T-CD63-GFP cells were transfected with RFP or NSP6-RFP. At 48 hpt, 10 µM MG-132 was added to treat cells for another 12 h. Cells were lysed and immunoprecipitated with anti-GFP beads. The expression levels of ubiquitin-conjugated proteins and GAPDH within both total and IP samples were subjected to Western blot assays. (**D**) NSP6 antagonized PSMD12 to enhance the ubiquitination of CD63 in HEK293T-CD63-GFP cells. HEK293T-CD63-GFP cells were transfected with PSMD12-RFP along with empty vector or NSP6-RFP, respectively. At 48 hpt, 10 µM MG-132 was added to the culture medium to treat cells for another 12 h. Cells were lysed and immunoprecipitated with anti-GFP beads. (**E**) PSMD12 reduced the ubiquitination levels of ACE2. HEK293T cells were transfected with ACE2-HA along with GFP or PSMD12-GFP, respectively. At 48 hpt, 10 µM MG-132 was added to treat the cell for 12 h. Cells were lysed and immunoprecipitated with anti-HA beads. (**F**) PSMD12 safeguarded the expression levels of ACE2. HEK293T cells were transfected and treated as in **E**. The expression levels of ACE2 and GAPDH within both total and IP samples were analyzed by Western blot. (**G**) PSMD12 reduced the lysine 48 (**K48**)-linked ubiquitination levels of CD63. HEK293T-CD63-GFP cells were co-transfected with Flag-Ub-K48O along with RFP or PSMD12-RFP, respectively. At 48 hpt, 10 µM MG-132 was added into the culture medium and incubated for 12 h. Cells were lysed and immunoprecipitated with anti-GFP beads. The expression levels of K48-linked ubiquitin-conjugated proteins and GAPDH within both total and IP samples were evaluated by Western blot assays. (**H**) PSMD12 reduced the K63-linked ubiquitination levels of CD63. HEK293T-CD63-GFP cells were co-transfected with Flag-Ub-K63O along with RFP or PSMD12-RFP, respectively. At 48 hpt, 10 µM MG-132 was added into the culture medium and incubated for 12 h. Cells were lysed and immunoprecipitated with anti-GFP beads. The expression levels of K63-linked ubiquitin-conjugated proteins and GAPDH within both total and IP samples were evaluated by Western blot assays. (**I**) PSMD12 reduced the K48-linked ubiquitination levels of ACE2. HEK293T-ACE2-GFP cells were co-transfected with Flag-Ub-K48O along with RFP or PSMD12-RFP, respectively. At 48 hpt, 10 µM MG-132 was added into the culture medium and incubated for 12 h. Cells were lysed and immunoprecipitated with anti-GFP beads. The expression levels of K48-linked ubiquitin-conjugated proteins and GAPDH within both total and IP samples were evaluated by Western blot assays. (**J**) PSMD12 reduced the K63-linked ubiquitination levels of ACE2. HEK293T-ACE2-GFP cells were co-transfected with Flag-Ub-K63O along with RFP or PSMD12-RFP, respectively. At 48 hpt, 10 µM MG-132 was added into the culture medium and incubated for 12 h. Cells were lysed and immunoprecipitated with anti-GFP beads. The expression levels of K63-linked ubiquitin-conjugated proteins and GAPDH within both total and IP samples were evaluated by Western blot assays.

To further identify the role of NSP6 and PSMD12 in the regulation of CD63, we conducted ubiquitination assays. The results demonstrated that PSMD12 exerted an inhibitory effect on the ubiquitination level of CD63 (Fig. 6A). Furthermore, we observed a concomitant increase in the expression level of CD63 in the presence of PSMD12, probably due to its ability to deubiquitinate CD63 and protect CD63 from degradation (Fig. 6B). We further verified whether NSP6 could promote the ubiquitination of CD63. We found that the ubiquitination level of CD63 was enhanced by NSP6, which could potentially explain NSP6-induced CD63 degradation (Fig. 6C). We also confirmed that NSP6 interfered with the deubiquitination of CD63 mediated by PSMD12. Results showed that the PSMD12-mediated ubiquitination of CD63 was enhanced by NSP6 (Fig. 6D). Furthermore, the impact of NSP6 on increasing the ubiquitination level of CD63 and interfering with the deubiquitination of CD63 by PSMD12 was also confirmed in Calu-3 cells (Fig. S7A and B). These results suggested that NSP6 could antagonize the deubiquitination of CD63 mediated by PSMD12, which kept the ubiquitination state of CD63 to promote its degradation. The effect of PSMD12 on ACE2 was also assessed. We found that PSMD12 also deubiquitinated ACE2, and the expression level of ACE2 was also enhanced by PSMD12 (Fig. 6E and F).

The K48- and K63-linked chains were identified as the two predominant linkage types in mammalian cells (81). The K48-linked chains serve as the most prevalent proteasomal targeting signals, whereas the K63-linked chains mainly regulate proteasome-independent events such as protein-protein interactions and endocytosis (82). To investigate the involvement of K48- or K63-linked polyubiquitination in PSMD12-mediated ubiquitination of CD63, we performed a ubiquitination assay using flag-tagged ubiquitin with only K48 (all lysines were mutated to arginines except K48, named Flag-Ub-K48O) and flag-tagged ubiquitin with only K63 (all lysines were mutated to arginines except K63, named Flag-Ub-K63O). We found that PSMD12 interrupted the K48- and K63-linked polyubiquitination of CD63 (Fig. 6G and H). The K48- and K63-linked polyubiquitination on ACE2 was also reduced by PSMD12 (Fig. 6I and J). These results indicated that that NSP6 might hijack the deubiquitination activity of PSMD12 on CD63 and ACE2, affected its role in proteasomal degradation, and interfered with protein-protein interactions between CD63, ACE2, and other potentially unknown host proteins, which disrupted the maintenance of CD63 and ACE2 stability by PSMD12 and promoted the degradation of CD63 and ACE2.

### The biogenesis of ACE2-exos was suppressed by NSP6 based on the SARS-CoV-2 replicon system

**Fig 7:**
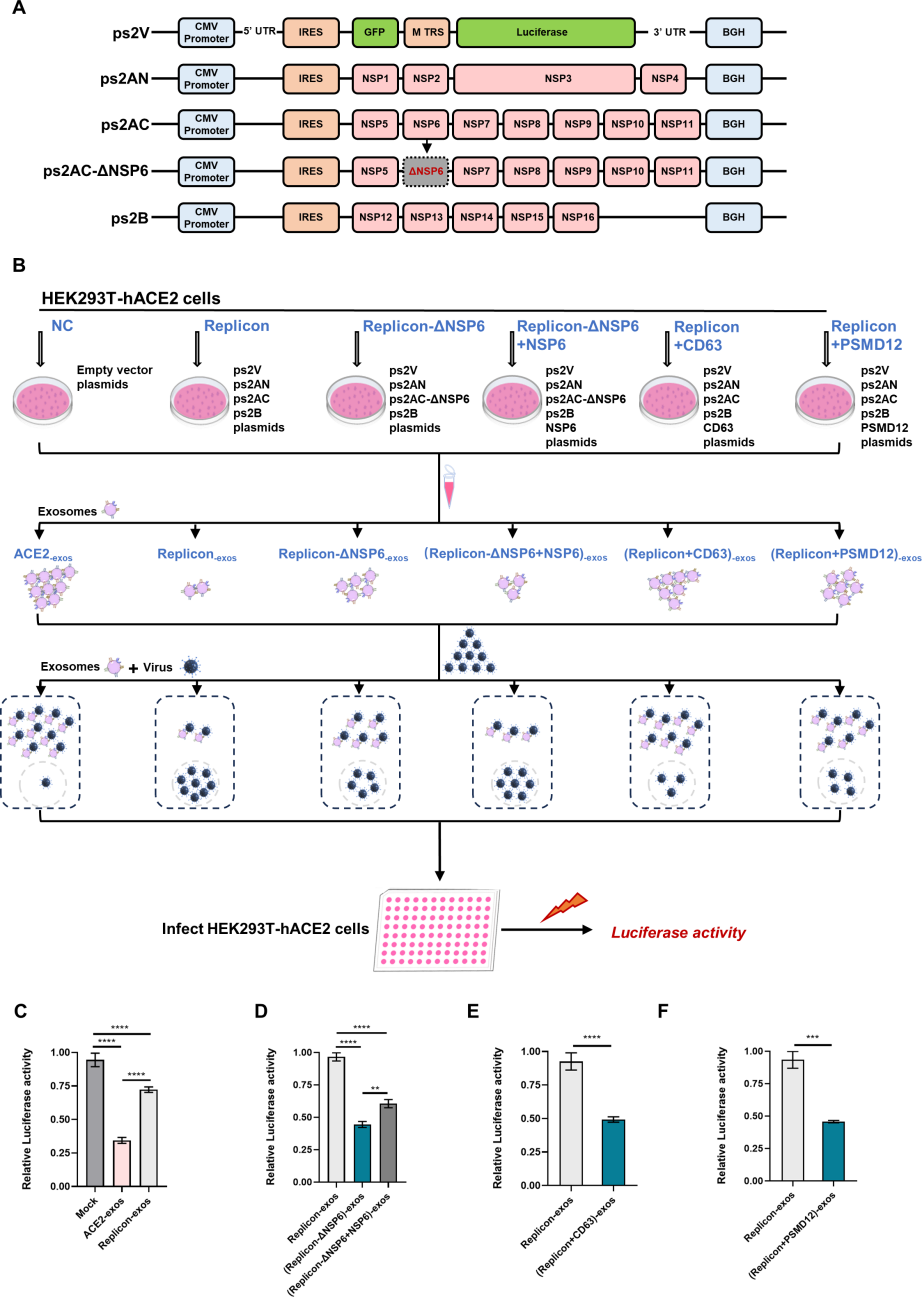
The biogenesis of ACE2-exos was suppressed by NSP6 based on the SARS-CoV-2 replicon system. (**A**) Schematic of the replicon system comprising ps2V, ps2AN, ps2AC, and ps2B. ps2AC-ΔNSP6 plasmid was constructed by removing the sequence of NSP6. (**B**) Schematic of the replicon system-based SARS-CoV-2 pseudotyped virus infection experiment. HEK293T-hACE2 cells were transfected with the indicated plasmids encoding replicon, replicon-ΔNSP6, co-transfected with Replicon-ΔNSP6 and NSP6, co-transfected with Replicon and CD63, or co-transfected with Replicon and PSMD12. The empty vector was transfected as negative control (NC). Supernatants of each group were collected and purified for exosomes at 24 hpt. Pseudotyped SARS-CoV-2 viruses were mixed with purified exosomes for 15 min at room temperature, followed by adding into HEK293T-hACE2 seeded into 96-well plates. The relative luciferase activities, which represented viral infectivity within different groups, were measured at 24 hpi. (**C**) The effect of replicon system on ACE2-exos blocking viral infection. (**D**) The effect of NSP6 on ACE2-exos blocking viral infection in the replicon system. (**E**) The effect of CD63 on ACE2-exos blocking viral infection in the replicon system. (**F**) The effect of PSMD12 on ACE2-exos blocking viral infection in the replicon system. The data were shown as mean ± SD (error bars) in triplicate. *P*-values were calculated by one-way ANOVA tests (**C and D**) or Student’s *t*-test (**E and F**). **P < 0.01, ***P < 0.001, ****P < 0.0001.

To further verify the effect of NSP6 on ACE2-exos and viral infection, we utilized a reliable SARS-CoV-2 replicon system developed in the previous study to assess the viral replication and the inhibitory effect of ACE2-exos (83). The replicon system consists of four plasmids expressing necessary viral genes and segments of SARS-CoV-2, including ps2V, ps2AN, ps2AC, and ps2B (Fig. 7A). The ps2AN expresses NSP1 to NSP4, the ps2AC expresses NSP5 to NSP11, and the ps2B expresses NSP12 to NSP16. The replicases and transcriptases were expressed from ps2AN, ps2AC, and ps2B plasmids. ps2V expresses the replicon RNA. The GFP serves as the indicator of transfection and transcription, while the luciferase gene acts as a reporter under the control of the M protein transcription regulating sequence.

To determine the role of NSP6, we also constructed the ps2AC-ΔNSP6 plasmid through deletion of the NSP6 gene (Fig. 7A). The schematic of NSP6 on ACE2-exos based on the replicon system was depicted in Fig. 7B. HEK293T cells were co-transfected with ps2V, ps2AN, ps2AC, and ps2B plasmids (replicon) or ps2V, ps2AN, ps2AC-ΔNSP6, and ps2B plasmids (replicon-ΔNSP6) (Fig. 7B). The exosomes derived from HEK293T-hACE2 cells which were co-overexpressed with replicon attenuated the blocking effect of ACE2-exos and promoted viral infection (Fig. 7C). Compared with the exosomes derived from HEK293T-hACE2 cells overexpressing replicon, the exosomes derived from HEK293T-hACE2 cells overexpressing replicon-ΔNSP6 enhanced the blocking effect and inhibited viral infection (Fig. 7D). While the exosomes derived from HEK293T-hACE2 cells with co-overexpression of replicon-ΔNSP6 and NSP6 re-suppressed the blocking effect (Fig. 7D). Exosomes derived from co-overexpressing CD63 with replicon or co-overexpressing PSMD12 with replicon could reduce viral infection compared to those from overexpressing replicon only (Fig. 7E and F). Collectively, based on the experiments to delete or rescue NSP6 in the replicon system, we found that the NSP6 protein exerted a negative effect in ACE2-exos-mediated blocking of viral infection, which inhibited the production of ACE2-exos and promoted viral infection.

## DISCUSSION

The development of effective antiviral drugs and vaccines has changed the course of the COVID-19 pandemic and saved countless lives. However, the persistently emerging SARS-CoV-2 variants remain a threat (84, 85). Exosomes produced by SARS-CoV-2-infected cells carry and deliver ACE2 to the extracellular environment, providing a strategy to avoid susceptible bystander cells being infected. Several studies have shown that ACE2-exos have superior antiviral properties in response to SARS-CoV-2 infection (54, 86). ACE2-based strategies, such as recombinant soluble ACE2 and ACE2-exos, have been proven to have broad neutralizing activities that can avoid viral mutation escape (55, 86, 87). ACE2-exos exhibited better efficacy than recombinant ACE2 in neutralizing SARS-CoV-2, which indicated great potential of ACE2-exos for future treatment (49). Many researchers are devoted to the study of ACE2-exos to improve its effectivity. A study showed that ACE2 was palmitoylated by zinc finger DHHC-type palmitoyltransferase 3 (ZDHHC3), which was critical for membrane-targeting and secretion of ACE2 into EVs (53). Our study showed that ACE2-exos could be upregulated by IFN-α/β and block the cell entry of SARS-CoV-2 (55). Given the roles played by ACE2-exos in antiviral activity, it is not surprising that pathogenic organisms could evolve strategies to regulate exosomes in order to evade host surveillance and enhance their virulence. Both the cell entry of SARS-CoV-2 and the production of exosomal hACE2 depend on endocytosis. Exosomes are part of the endocytic system, which are tightly regulated and able to respond to several stimuli and lead to alterations in the composition.

To explore whether viral proteins could regulate ACE2-exos against host defense, we screened for viral proteins that might influence exosome production. As the first characterized tetraspanin, CD63 is abundant in exosomes and is considered to be the most classical exosome biomarker (37, 57). Besides, CD63 is widely distributed in endosomes, including early endosomes, late endosomes, and MVBs. Therefore, we choose CD63 as the main indicator of exosomes. First, we found that NSP6 is involved in the biogenesis of exosomes. NSP6 downregulated the level of intracellular CD63 in a dose-dependent manner. Given that CD63 participated in the formation of exosomes, we believed that NSP6-mediated CD63 downregulation might influence exosome production, cargo sorting, or vesicle transport. By overexpression or knockout of CD63, we confirmed the effect of CD63 on exosomes. We further confirmed that NSP6 decreased the number of exosomes by downregulating CD63. Second, we confirmed the reduction of ACE2-exos under the NSP6 regulation. We confirmed that the amounts of ACE2 proteins secreted into the extracellular environment were reduced by two factors: the decrease of total exosomes and the restriction of ACE2 sorted into exosomes. Third, NSP6 promoted viral infection by diminishing the blocking effect of ACE2-exos. NSP6 promoted the infection of SARS-CoV-2 to bystander healthy cells, which was consistent with the effect of deletion CD63. In contrast, overexpression of CD63 rescued the secretion of ACE2-exos and enhanced the blocking effect of viral infection. It has been reported that overexpression of tetraspanin CD9 also enhanced the production of EVs (52, 88). It is reasonable that CD63 enhanced exosome production to promote ACE2 secretion.

Several studies have identified the role of PSMD12 in the maintenance of cellular protein homeostasis. PSMD12 can abate the ubiquitination level of cyclin-dependent kinase inhibitor 3 (CDKN3), thereby stabilizing CDKN3 and promoting pancreatic cancer progression (66, 89). Our results showed that PSMD12 interacted with CD63 and ACE2 to promote their deubiquitination and avoid degradation. Meanwhile, NSP6 bound to PSMD12 to restrict its activity, resulting in impeding the deubiquitination of CD63, promoting CD63 degradation, and reducing the production of ACE2-exos. We observed that both K48- and K63-linked polyubiquitination of CD63 and ACE2 were disrupted by PSMD12. It was well known that K48-linked chains target proteins for proteasomal degradation, whereas K63-linked chains regulate functions of target proteins, including protein-protein interactions, intracellular localization, DNA repair, and endocytosis (90). Notably, a study found that K63-linked ubiquitination promotes proteasomal degradation by seeding branched chains (91). The involvement of K48-linked polyubiquitination suggests that NSP6 may affect the safeguarding role of PSMD12 in proteasomal degradation, thereby promoting the degradation of CD63 and ACE2. The involvement of K63-linked polyubiquitination indicates that NSP6 may modulate protein-protein interactions between CD63, ACE2, and other unknown host proteins to disrupt their biological functions.

**Fig 8:**
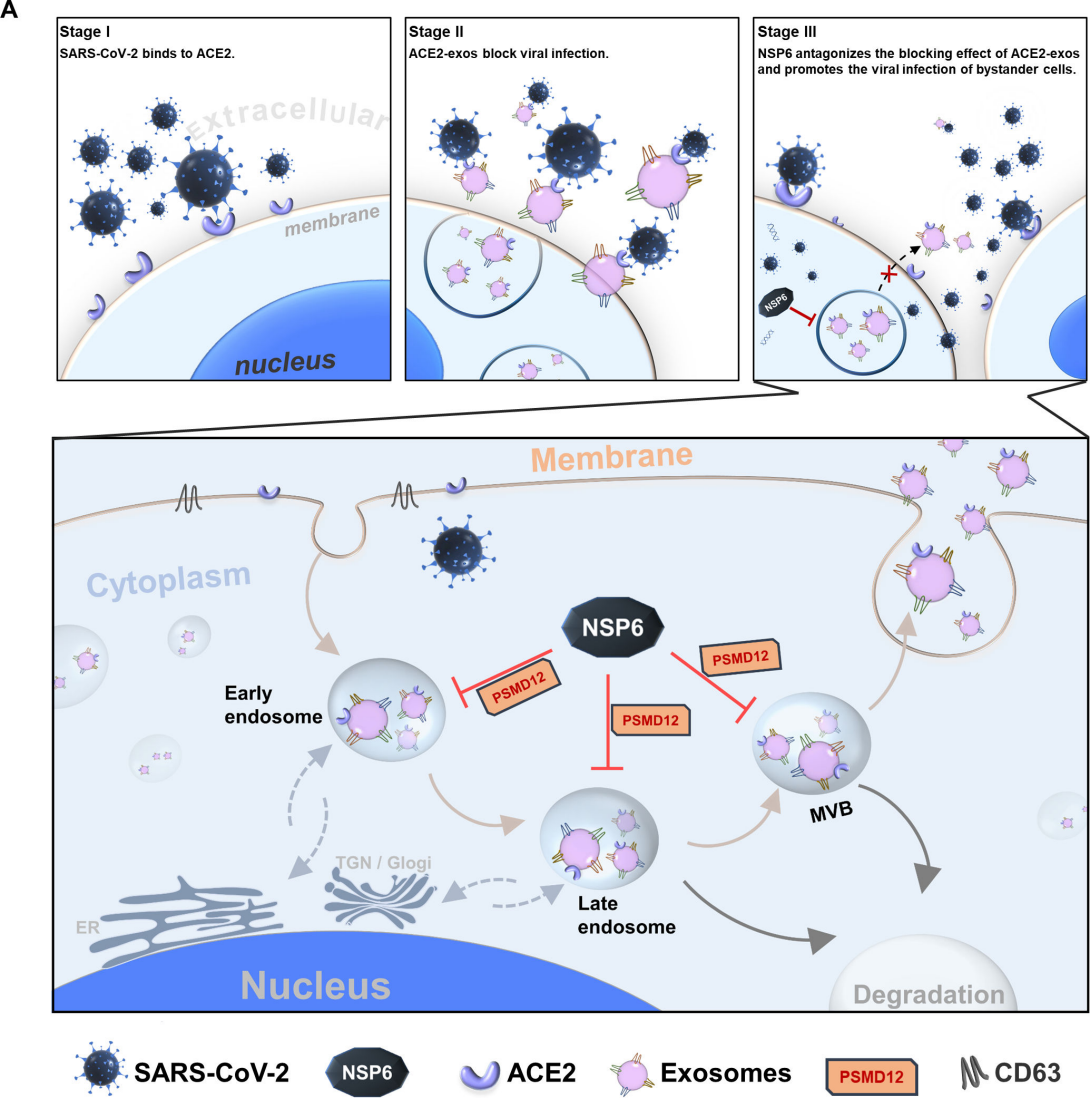
Schematic of NSP6 hijacking the biogenesis of ACE2-exos. (**A**) SARS-CoV-2 infected host cells mainly by binding to ACE2 on the cell surface (stage I). Upon SARS-CoV-2 infection, ACE2-containing exosomes (ACE2-exos) could be induced to bind to free virions and avoid susceptible bystander cells being infected (stage II). NSP6 inhibited the production of ACE2-exos and facilitated viral infection to adjacent cells (stage III). CD63 was involved in all the biogenesis stages of exosomes, including early endosome (EE) formation, later endosome (LE) maturation, multivesicular body (MVB) formation, and exosome secretion. CD63 positively regulated the production of ACE2-exos. While in SARS-CoV-2-infected cells, viral protein NSP6 promoted CD63 degradation within EE, LE, and MVB throughout the endocytic pathway by enhancing its ubiquitination, thereby inhibiting the production of total exosomes as well as ACE2-exos. PSMD12 was involved in maintaining the homeostasis of CD63 and ACE2, which was hijacked and antagonized by NSP6. The inhibition of the production of ACE2-exos by NSP6 eventually promoted free virions to infect healthy bystander cells.

Finally, a biosafety replicon system was employed to validate the role of NSP6 on ACE2-exos in blocking viral infection. The deletion of NSP6 significantly suppressed viral infection of bystander cells, possibly due to an increase in ACE2-exos. Collectively, considering the remarkable antiviral efficacy of ACE2-exos, we propose a model of NSP6-mediated viral infection by inhibiting the production of ACE2-exos. Once SARS-CoV-2 binds to ACE2 on the cell surface to infect host cells, ACE2-exos are generated as a defense mechanism against viral invasion. However, NSP6 antagonizes the blocking effect of ACE2-exos by binding to PSMD12 to restrict its deubiquitination activity on CD63 and ACE2, which ultimately inhibits the production of ACE2-exos by promoting the degradation of CD63 and ACE2 (Fig. 8A).

A limitation of our study is that the regulatory roles of viruses in the biological process, especially in the sorting and trafficking of functional proteins such as ACE2, remain elusive due to the diversity and complex localization of key proteins involved in the formation of exosomes. NSP6 may interact with multiple proteins at multiple stages to interfere with the biogenesis of ACE2-exos. Whether NSP6 could hijack specific E3 ubiquitin ligases to facilitate the degradation of CD63 and ACE2 still needs to be clarified. Besides, we only elucidated the roles of NSP6 on ACE2-exos for pseudotyped SARS-CoV-2 variants. Further authentic SARS-CoV-2 infection studies need to be conducted to decipher NSP6-mediated enhancement of viral infection fully. The key to blocking SARS-CoV-2 infection lies in understanding the virus-host interaction, which is a complex process involving multiple proteins at multiple stages. NSP6 is a complex multi-transmembrane protein, the structure of which has not been resolved. In the future, the resolving of the crystal structure of NSP6 and corresponding co-crystallization structures with interacting proteins will provide crucial insight into its molecular mechanism and facilitate the development of targeted drugs.

Previous studies have shown that the proportion of ACE2-positive EVs, as well as the amount of ACE2 displayed on exosomes, varied from BALF samples of critically ill COVID-19 patients (52). Patients with high amounts of ACE2-positive exosomes in their BALFs were hospitalized for a shorter duration. Our results demonstrated that NSP6 effectively reduced the expression level of ACE2-exos, which might be the cause of the phenomenon. Circulating exosomes in COVID-19 patients may modulate immune response, inflammation, and coagulation pathways (92). The regulation of NSP6 to ACE2-exos is likely to affect pathologically relevant clinical indices significantly. Deciphering the mechanisms of virus-cell-EV interaction may facilitate the design of EVs that inhibit viral infection and enable targeted therapy. We believe that targeting NSP6 may be a potential strategy for enhancing the blocking effect of ACE2-exos to accelerate the recovery of COVID-19 patients further.

## MATERIALS AND METHODS

### Cell culture and transfection

HEK293T, A549, and Calu-3 cell lines were purchased from the American Type Culture Collection. HEK293T, HEK293T-hACE2, HEK293T-sgCD63, HEK293T-CD63-GFP, HEK293T-NSP6, A549, and A549-NSP6 cells were cultured in Dulbecco’s modified Eagle’s medium (DMEM) supplemented with 10% fetal bovine serum (FBS) or exosome-depleted FBS (Systembio, EXO-FBS-50A-1), 1% penicillin-streptomycin. Calu-3 and Calu-3-NSP6 cells were cultured in minimum essential medium Eagle with Earle’s salts and L-glutamine (MEM). All cells were maintained in a humidified incubator at 37℃ with 5% CO_2_. All cells have been confirmed to be mycoplasma free by PCR-based assay. Cells were transfected with the indicated plasmids using Lipofectamine 2000 according to the manufacturer’s instructions.

### Reagents and antibodies

DMEM, FBS, and penicillin-streptomycin were obtained from Gibco. MEM was purchased from Corning. Lipofectamine 2000 and 4′,6-diamidino-2-phenylindole dihydrochloride (DAPI) were obtained from Thermo Fisher Scientific. MG132 was purchased from Selleck. All the pools of siRNA were obtained from the RiboBio Company (Guangzhou, China). The antibodies used for flow cytometry included PE-conjugated antibodies against CD63 (PE-anti-human CD63) (353003, BioLegend), APC-anti-human CD9 (312107, BioLegend), and APC-anti-human CD81 (349509, BioLegend). The antibodies used for Western blot included rabbit-anti-ubiquitin (10201-2-AP, Proteintech), mouse-anti-RFP-Tag (T0055, Affinity), rabbit-anti-ACE2 (ab108252, Abcam), mouse-anti-calnexin (66903-1-IG, Proteintech), rabbit-anti-CD63 (25682-1-AP, Proteintech), rabbit-anti-HA (51064-2-AP, Proteintech), rabbit-anti-GFP-Tag (50430-2-AP, Proteintech), mouse-anti-GAPDH (60004-1-Ig, Proteintech), and rabbit-anti-PSMD12 (39119, Signalway Antibody). The secondary antibodies used for developing targeted proteins in Western blotting assays included IRDye 680RD Goat anti-Mouse (926-68070) and IRDye 800CW Goat anti-Rabbit (926-32211).

### Plasmids and constructs

The DNA sequences of SARS-CoV-2 viral proteins were chemically synthesized in GENEWIZ and inserted into pcDNA3.1 vectors. The pCMV3-Flag-Rab7, pCMV3-Rab5-Myc, pCMV3-Rab27A-GFP, pcMV3-Rab27B-GFP, and pCMV3-PSMD12-GFP were purchased from Sino Biological. The RFP coding sequence was constructed into the pcDNA3.1 vector, named pcDNA3.1-RFP vector. The 3× HA coding sequence was constructed into the pcDNA3.1 vector, named pcDNA3.1-3× HA vector. The GFP coding sequence was constructed into the pSin-EF2-puro-oligo vector, named the pSin-GFP vector. The sequences of NSPs (NSP1-16) and ORFs (ORF3, 6, 7, 8, and 10) were amplified with PCR and inserted into a mammalian expression vector with a GFP tag, named NSP (1–16)-GFP and ORF (3, 6, 7, 8 and 10)-GFP, respectively. The typical PLpro domain (746–1,059 amino acid) of NSP3 was selected for the construction of NSP3-GFP due to the large molecular weight of full-length NSP3 (93). The sequence of NSP6 was amplified with PCR and inserted into pcDNA3.1-3× HA, pcDNA3.1-RFP, and pEGFP-N1 vector, named NSP6-HA, NSP6-RFP, and NSP6-GFP, respectively. The sequence of NSP6 with a C-terminal HA tag was amplified with PCR and inserted into pSin-EF2-puro-oligo, named pSin-NSP6-HA. The sequence of CD63 was amplified with PCR and inserted into pcDNA3.1-3× HA, pcDNA3.1-RFP, pEGFP-N1, and pSin-GFP vector, named CD63-HA, CD63-RFP, CD63-GFP, and psin-CD63-GFP, respectively. The sequence of PSMD12 was amplified with PCR and inserted into pcDNA3.1-RFP, named PSMD12-RFP. The sequences of Rab5 and Rab7 were also amplified with PCR and inserted into pcDNA3.1-RFP or pEGFP-N1 vectors to construct the GFP- or RFP-tagged plasmids. All constructs were verified by Sanger DNA sequencing.

### Generation of stable cell lines

HEK293T-NSP6, A549-NSP6, and Calu-3-NSP6 cell lines were generated as follows. Lentiviral plasmids pSin-NSP6-HA were transfected into HEK293T cells together with psPAX2 and VSV-G. The supernatants were collected at 48 h post transfection. The viruses were concentrated by PEG 6000. HEK293T, A549, and Calu-3 cells were infected with these lentiviruses to produce HEK293T-NSP6, A549-NSP6, and Calu-3-NSP6 cell lines, respectively. Positive clones were selected by 3 µg/mL puromycin at 48 h post-transfection.

The HEK293T-CD63-GFP cell line was generated as follows. Lentiviral plasmids pSin-CD63-GFP were transfected into HEK293T cells together with psPAX2 and VSV-G. The supernatants were collected at 48 h post transfection. The viruses were concentrated by PEG 6000. HEK293T cells were infected with the lentiviruses to generate HEK293T-CD63-GFP cell lines. Positive clones were selected by 3 µg/mL puromycin at 48 h post-transfection.

The HEK293T-sgCD63 cell line was generated as follows. Oligonucleotides of sgRNA against human CD63 included sgCD63-1: 5′-CAACCACACTGCTTCGATCC-3′, sgCD63-2: 5′-GAGGTGGCCGCAGCCATTGC-3′, and sgCD63-3: 5′-CTTTCTGTCTCTTATCATGT-3′. The sequences were cloned into the lentiCRISPRv2.0 vector (Addgene plasmid #52961). The resulting lentiviral plasmids were transfected into HEK293T cells together with psPAX2 and VSV-G. The supernatants were collected at 48 h post transfection. The viruses were concentrated by PEG 6000. Cells were infected with these lentiviruses. Positive clones were selected by 3 µg/mL puromycin at 48 h post transfection. The most efficient one (sgCD63-3: CTTTCTGTCTCTTATCATGT) was chosen and proceeded to conduct all the knockout experiments.

### Real-time quantitative PCR (RT-qPCR)

Total RNA was extracted with an EZ-press RNA Purification Kit (B0004D, EZBioscience) according to the manufacturer’s instructions. Reverse transcription was conducted with 5× HiScript III qRT SuperMix (R323-01-AC, Vazyme), and then, quantitative PCR was performed using ChamQ Universal SYBR qPCR Master Mix (Q711-02, Vazyme). The mRNA levels of target genes were normalized to GAPDH. The primer sequences used for RT-qPCR were as follows: GAPDH forward primer: 5′-TGACTTCAACAGCGACACC-3′; GAPDH reverse primer: 5′-CACCCTGTTGCTGTAGCCAAA-3′; CD63 forward primer: 5′-GGACAGGATGCAGGCAGATT-3′; CD63 reverse primer: 5′-TTAATGCAGCAGGAGTCGGG-3′; Luciferase forward primer: 5′-GTGCAGCGAGAATAGCTTGC-3′; Luciferase reverse primer: 5′-TTGCTCACGAATACGACGGT-3′.

### Immunofluorescence assay and structured illumination microscopy (SIM) imaging

HEK293T cells were seeded in u-slide chambered coverslips (Ibidi, 80826), which were pre-treated with poly-lysine (Sigma-Aldrich). IF assay was performed as previously described (94). Briefly, cells were fixed with 4% poly-formaldehyde at room temperature for 10 min, then permeabilized with 1% Triton X-100 in phosphate buffered saline (PBS) for 10 min, followed by washing three times with PBS. Cells were blocked with 5% BSA in PBS for 45 min, followed by washing three times with PBST (PBS with 0.1% Tween-20). Blocked samples were subjected to sequentially incubate with specific primary antibodies and fluorescently labeled secondary antibodies at room temperature for 45 min. After incubation, cells were washed with PBST three times. Cells were treated with a DAPI solution at room temperature for 10 min, followed by washing three times with PBST. Finally, the samples were stored at 4°C or imaged with super-resolution SIM. Prepared samples were imaged on an Eclipse Ti inverted microscope. The original images were acquired and reconstructed to form the SIM image. All the SIM images were analyzed with the N-SIM module of the NIS-Elements AR software (Nikon).

### Western blot assay

Western blot assay was performed as described previously (55, 95). Briefly, cells or purified exosomes were lysed in NP-40 lysis buffer (10 mM Tris-HCl buffered at pH 7.5, 150 mM NaCl, 0.5% NP-40, 1% Triton X-100, 10% glycerol, 2 mM EDTA, 1 mM NaF, 1 mM Na_3_VO_4_ and 1% protease inhibitor mixture). The cell lysates were separated by centrifugation at 12,000 rpm for 10 min at 4°, and the concentration was measured by the Bradford method. The lysates were separated by SDS-PAGE and transferred onto nitrocellulose membranes. The membranes were further blocked with 5% non-fat dry milk at room temperature for 1 h, followed by sequentially incubating with primary antibodies and secondary antibodies. The antibodies-incubated membranes were visualized with Odyssey CLX Imager (LI-COR Biosciences) and analyzed by Image Studio Lite Ver 4.0 (LI-COR Biosciences).

### Co-immunoprecipitation

Co-IP assay was performed as previously described (5, 94). HA-tagged or GFP-tagged protein constructs were transfected into HEK293T cells. At 48 h post transfection, cells were harvested and lysed in NP-40 lysis buffer. Anti-HA-tag or anti-GFP-tag beads were washed five times with ice-cold STN buffer (10 mM Tri-HCl buffered at pH 7.4, 150 mM NaCl, 0.5% NP-40, and 0.5% Triton X-100). Cell lysates were incubated with prepared anti-HA or anti-GFP beads for 4 h or overnight at 4°C while rotating. Then, the immunoprecipitates, which contained co-immunoprecipitated proteins, were washed five times with ice-cold STN buffer, eluted by boiling with 5× protein SDS-PAGE loading buffer at 100°C for 10 min and separated by SDS-PAGE for Western blotting or mass spectrometry analysis.

### Mass spectrometry

HEK293T cells were seeded into a 6-cm dish and transfected with 4 µg of NSP6-GFP plasmid. At 48 h post transfection, cells were collected and lysed for Co-IP assay. The separated proteins were then visualized with ProteoSilver Plus Silver Stain Kit (Sigma-Aldrich) according to the manufacturer's instructions. The whole lane of each sample was cut into nine slices and prepared for liquid chromatography–tandem mass spectrometry analysis as previously described (94). Functional pathways were analyzed from the gene ontology biological processes database (Gene Ontology Consortium) using DAVID Bioinformatics Resources.

### Purification and characterization of exosomes

Cells were cultured in media supplemented with 10% exosome-depleted FBS. Supernatants from cells cultured for 24 h were collected. Exosomes were purified with a differential ultracentrifugation as described previously (55). The supernatants were successively centrifuged at 300 × *g* for 10 min, 2,000 × *g* for 10 min, and 10,000 × *g* for 30 min at 4°C. The supernatants were filtered through a 0.22-µm filter and ultracentrifuged at 140,000 × *g* for 70 min at 4°C, followed by one wash with PBS. The final supernatants were ultracentrifuged again at 140,000 × *g* for 70 min at 4°C to obtain exosomes. TEM was used to characterize the successful isolation of exosomes. As previously described, exosomes were dropped onto the copper mesh for 10 min, and excess liquid was removed by filter paper. The copper mesh was dropped with 2% phosphotungstic acid for 3 min, and the excess liquid was removed by filter paper. After the copper mesh dried at room temperature, the exosomes were imaged with an HT7700 transmission electron microscope (Hitachi). iEM was used to characterize ACE2-containing exosomes. For immunogold labeling, purified exosomes were dropped onto the nickel grids for 20 min, blocked with 5% BSA for 10 min, and incubated with anti-ACE2 primary antibodies, followed by incubating with the protein A-gold particle-conjugated anti-Rabbit secondary antibodies. Finally, the exosomes were fixed with 1% glutaraldehyde for 5 min, stained with phosphotungstic acid for 5 min, and allowed to dry before imaging. The size and concentration of exosomes were analyzed by Nano-FCM (Flow NanoAnalyzer U30E).

### Pseudotyped virus infection assay

SARS-CoV-2 S/HIV-1 pseudotyped viruses were packaged by co-transfecting with SARS-CoV-2 S-expressing plasmids, pHIV-luciferase constructs, and psPAX2 plasmids. The supernatants were collected at 48 h post transfection, filtered through a 0.45-µm pore-size filter, and stored at −80°C until using. To test the blocking effect of ACE2-exos on viral infection, HEK293T-hACE2 cells or A549-hACE2 cells were pre-seeded into 96-well plates. Exosomes were mixed with pseudotyped viruses at a volume ratio of 1:1 and incubated at room temperature for 15 min. The supernatant of the 96-well plate was then replaced with 200 µL of the exosomes-viruses mixture. Infected cells were harvested at 24 h post infection and lysed with 50-µL 5× Passive Lysis Buffer (E194A, Promega). The luciferase activity was analyzed with a Spark-Multifunctional Microplate Detector.

### Statistical analysis

Data were analyzed utilizing GraphPad 7.0 software (La Jolla, CA, USA). The two-tailed Student's *t*-test and one-way analysis of variance tests were used to determine the significance of statistical data. All the experiments were repeated at least three times, and data were shown as the mean ± SD of three independent experiments and considered significant at *P < 0.05, **P < 0.01, ***P < 0.001, and ****P < 0.0001.

## Data Availability

The data used and/or analyzed to support the findings of this study are available in this paper or in the supplemental information. Any other raw data that support the findings of this study are available from the corresponding authors upon reasonable request.

## SUPPLEMENTAL MATERIAL

Supplemental figures mbio.03358-23-s0001.docx Fig. S1 to S7.

ASM does not own the copyrights to Supplemental Material that may be linked to, or accessed through, an article. The authors have granted ASM a non-exclusive, world-wide license to publish the Supplemental Material files. Please contact the corresponding author directly for reuse.
