# Supplementary material for: NSP6 inhibits the production of ACE2-containing exosomes to promote SARS-CoV-2 infectivity
Source: mBio. 2024 Feb 2;15(3):e03358-23. doi: 10.1128/mbio.03358-23 (PMC10936183; doi:10.1128/mbio.03358-23)
Supplement: Supplemental figures — Fig. S1 to S7. [file mbio.03358-23-s0001.docx]

## Supplementary Figures

**Figure S1**


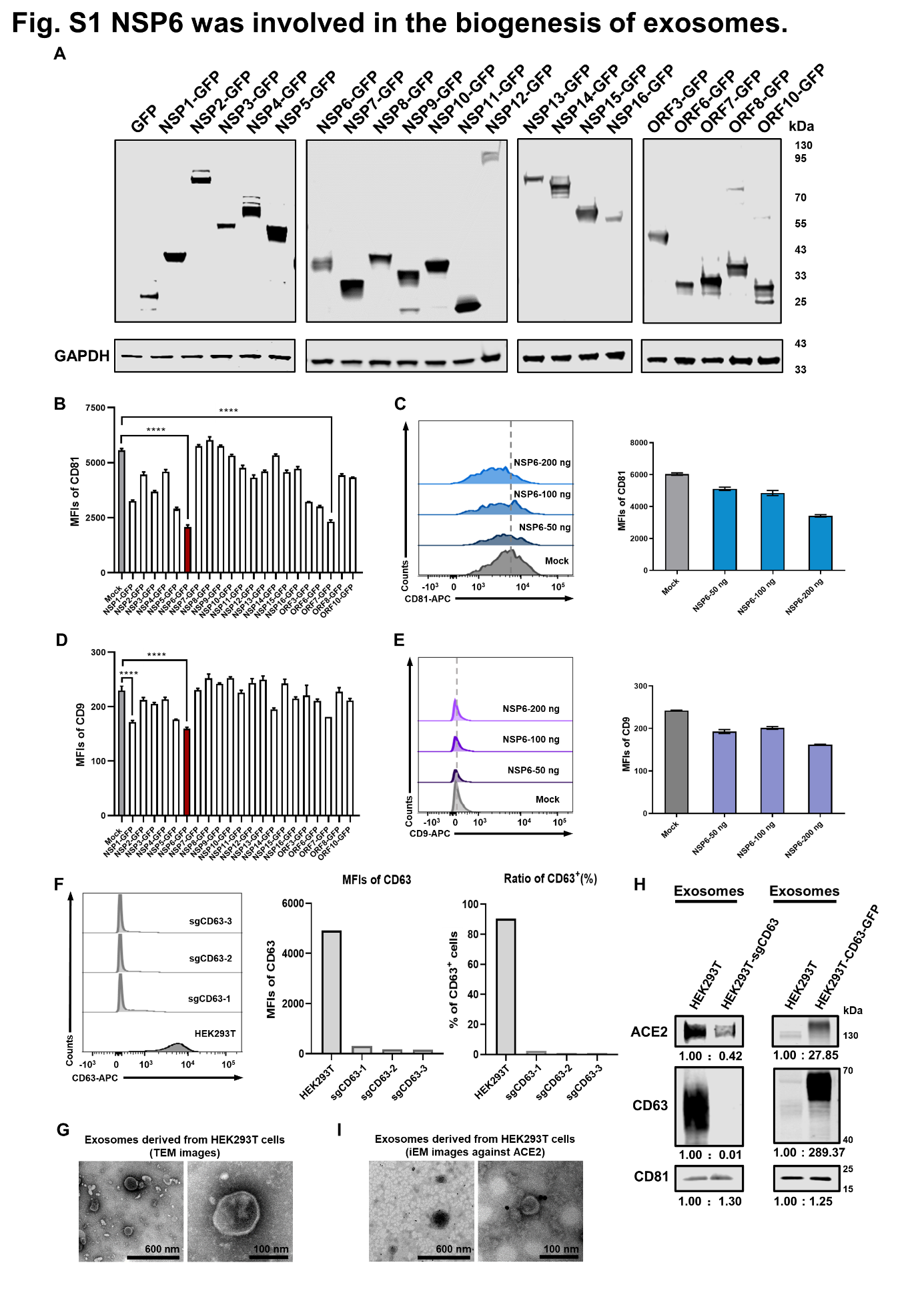


**Figure S1. NSP6 was involved in the biogenesis of exosomes.**

**(A)** Expression of SARS-CoV-2 viral proteins. HEK293T cells were transfected with 300 ng of empty vector (GFP group) or GFP-tagged SARS-CoV-2 protein-expressing plasmids respectively, followed by western blot against GAPDH (Internal control) and GFP. **(B)** HEK293T cells were transfected with 300 ng of empty vector (Mock group) or GFP-tagged SARS-CoV-2 protein-expressing plasmids, respectively. Cells were collected at 48 h post-transfection, mean fluorescence intensities (MFIs) of CD81 were analyzed by flow cytometry, gated on GFP^+^ cells. **(C)** HEK293T cells were transfected with various amounts (50 ng, 100 ng, and 200 ng) of NSP6-GFP plasmids, and MFIs of CD81 were analyzed by flow cytometry, gated on GFP^+^ cells. **(D)** MFIs of CD9 cells were analyzed by flow cytometry. The assay was performed as in **(B)**. **(E)** HEK293T cells were transfected as in (**C**). MFIs of CD9 were analyzed by flow cytometry, gated on GFP^+^ cells. **(F)** The knockout efficiencies of CD63 in HEK293T-sgCD63 were verified by flow cytometry. MFIs of CD63 and ratios of CD63-positive (CD63^+^) cells within different groups were plotted. **(G)** Representative transmission electron microscopy (TEM) images of exosomes purified from HEK293T cells. Scale bars represented 600 nm (primary amplification, left) and 100 nm (secondary amplification, right). **(H)** Western blot analysis of purified exosomes which derived from HEK293T, HEK293T-sgCD63, and HEK293T-CD63-GFP cells was performed. **(I)** Representative immune-electron microscopy (iEM) images of exosomes purified from HEK293T cells. Samples were immunogold-labeled with anti-ACE2 antibodies. Black dots indicated 10 nm gold particles. Scale bars represented 600 nm (primary amplification, left) and 100 nm (secondary amplification, right). The data were shown as mean ± SD (error bars) in triplicate. *P*-values were calculated by one-way ANOVA test (**B**-**E**). **** *P* < 0.0001.

**Figure S2**


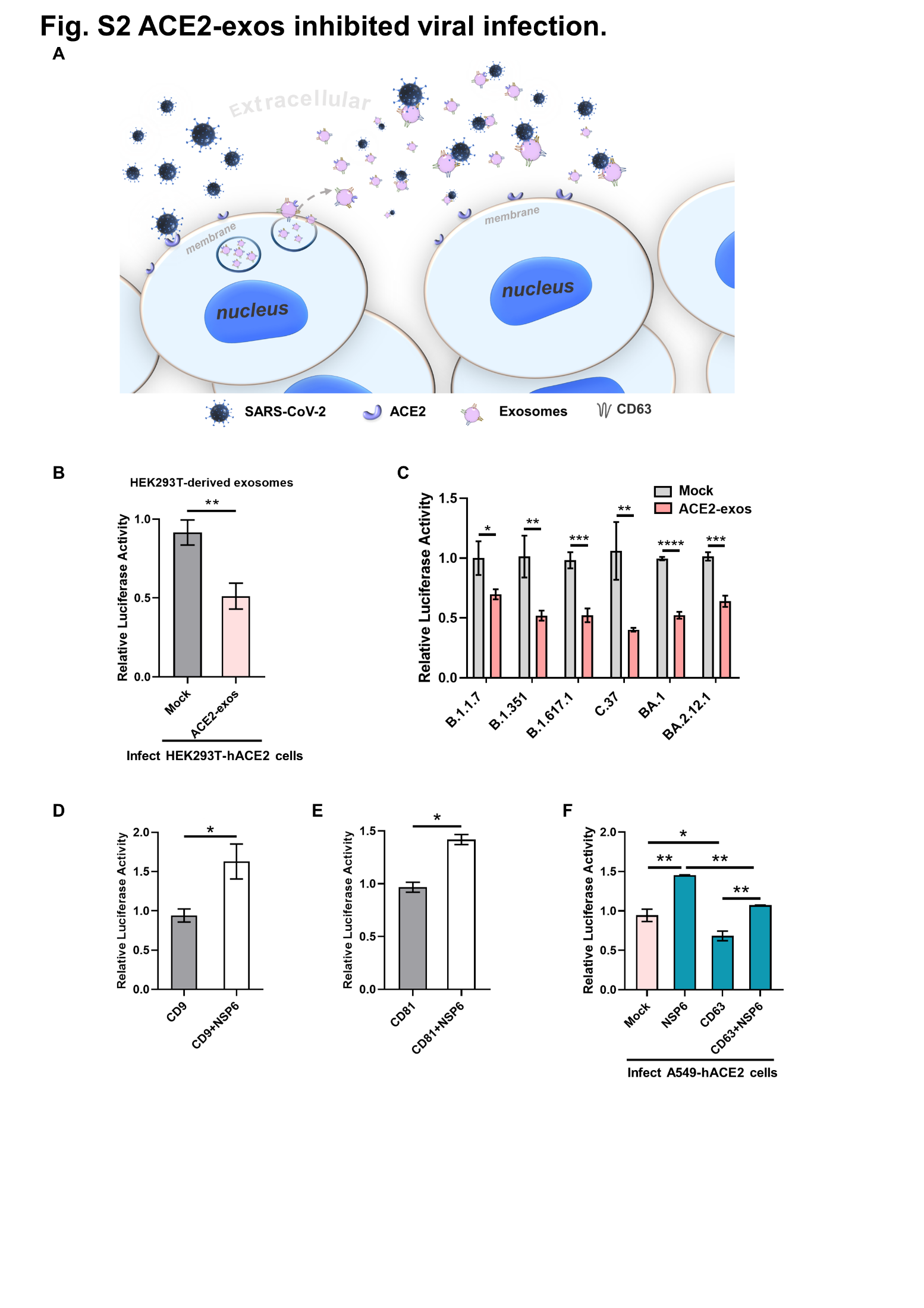


**Figure S2. ACE2-exos inhibited viral infection.**

**(A)** Schematic of ACE2-exos blocking viral infection to adjacent cells. **(B)** Exosomes derived from HEK293T cells, which were transfected with empty vector (Mock group) and ACE2-encoding plasmid (ACE2-exos group) respectively, were purified and mixed with pseudotyped viruses for 15 min at room temperature, followed by adding into HEK293T-hACE2 cells which were seeded into 96-well plates. The relative luciferase activities, which represented viral infectivity within different groups, were measured at 24 hpi. **(C)** The effect of ACE2-exos on the infection of pseudotyped SARS-CoV-2 variants. Exosomes derived from HEK293T-hACE2 cells (ACE2-exos group) were obtained and mixed with pseudotyped viruses, including B.1.1.7 (Alpha), B.1.351 (Beta), B.1.617.1 (Kappa), C.37 (Lambda), BA.1 (Omicron subvariant), and BA.2.12.1 (Omicron subvariant) for 15 min at room temperature, and added into HEK293T-hACE2 cells seeded into 96-well plates. Cells were treated with pseudotyped viruses-only groups were set as the Mock groups. **(D)** Exosomes were purified from HEK293T-hACE2 cells, which were transfected with CD9-expressing plasmid or co-transfected with CD9- and NSP6-expressing plasmids. The effects of these exosomes on pseudotyped virus infection were evaluated by luciferase assays. **(E)** Exosomes were purified from HEK293T cells, which were transfected with CD81-expressing plasmid or co-transfected with CD81- and NSP6-expressing plasmids. The effects of these exosomes on pseudotyped virus infection were evaluated by luciferase assays. **(F)** The effect of ACE2-exos derived from HEK293T-hACE2 cells overexpressed with NSP6, CD63, or co-overexpressed with CD63 and NSP6 on viral infection within A549-hACE2 cells. Pseudotyped SARS-CoV-2 viruses were co-incubated with NSP6-overexpressing ACE2-exos (NSP6), CD63-overexpressing ACE2-exos (CD63), or CD63 and NSP6 co-overexpressing ACE2-exos (CD63+NSP6), followed by infecting A549-hACE2 cells. The relative luciferase activities within each group were analyzed at 24 hpi. These data were shown as mean ± SD (error bars) in triplicate. *P*-values were calculated by Student's *t*-test (**B**-**E**) or one-way ANOVA tests (**F**). * *P* < 0.05, ** *P* < 0.01, *** *P* < 0.001, **** *P* < 0.0001.

**Figure S3**


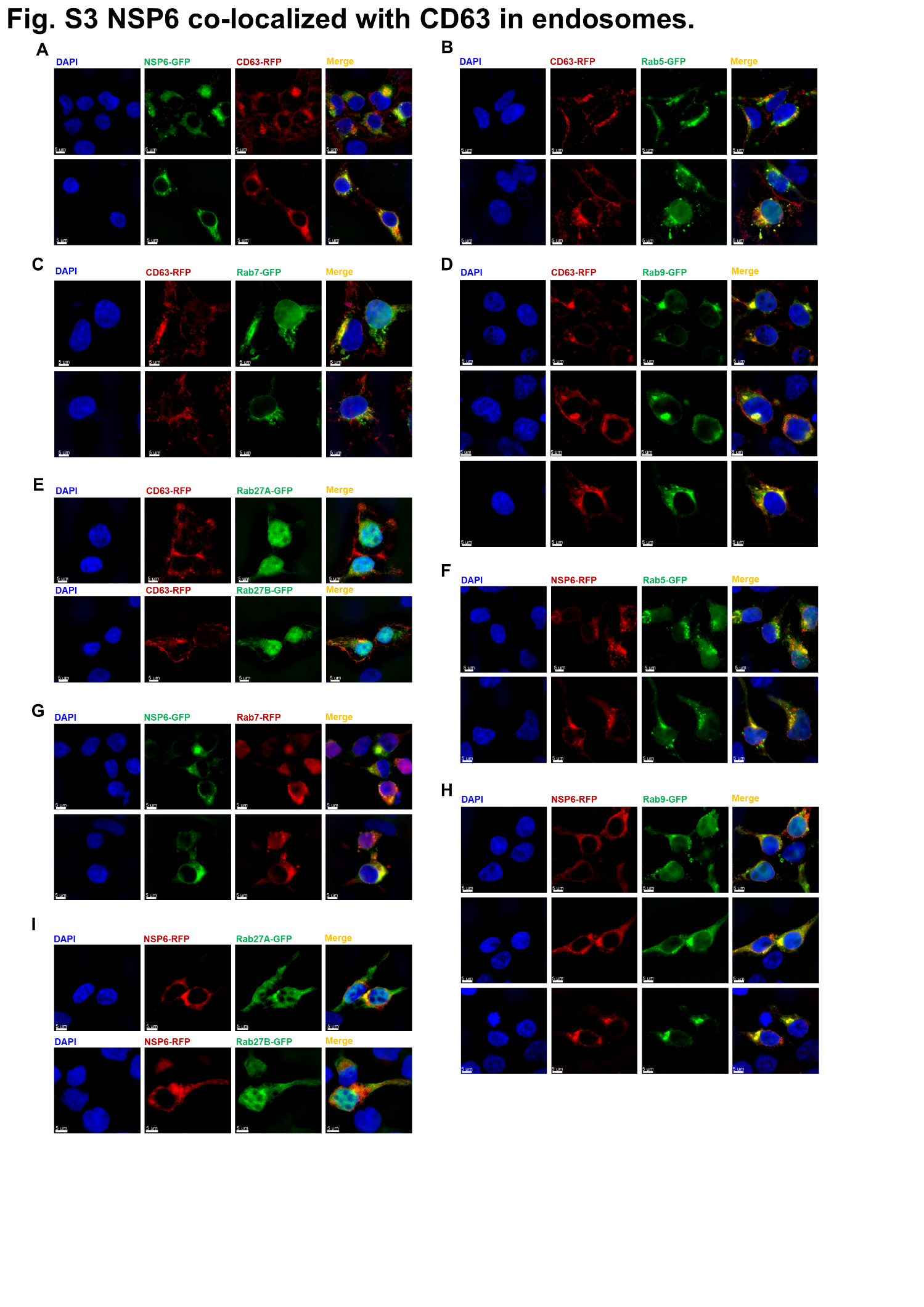


**Figure S3. NSP6 co-localized with CD63 in endosomes.**

**(A)** The localization of NSP6 and CD63 within HEK293T cells. Cells were transfected with NSP6-GFP, CD63-RFP, or co-transfected with NSP6-GFP and CD63-RFP. Cells were harvested for conducting immunofluorescence (IF) assay at 48 hpt. **(B)** The co-localization of CD63 with Rab5 in HEK293T cells. Cells were transfected with CD63-RFP along with Rab5-GFP. Cells were subjected to IF assay at 48 hpt. **(C)** The co-localization of CD63 with Rab7 in HEK293T cells. Cells were transfected with CD63-RFP along with Rab7-GFP. Cells were subjected to IF assay at 48 hpt. **(D)** The co-localization of CD63 with Rab9 in HEK293T cells. Cells were transfected with CD63-RFP along with Rab9-GFP, followed by IF assays at 48 hpt. **(E)** The co-localization of CD63 with Rab27A and Rab27B in HEK293T cells. Cells were transfected with CD63-RFP along with Rab27A-GFP or Rab27B-GFP respectively, followed by IF assays at 48 hpt. **(F)** The co-localization of NSP6 with Rab5 in HEK293T cells. Cells were transfected with NSP6-RFP along with Rab5-GFP. Cells were subjected to IF assay at 48 hpt. **(G)** The co-localization of NSP6 with Rab7 in HEK293T cells. Cells were transfected with NSP6-GFP along with Rab7-RFP. Cells were subjected to IF assay at 48 hpt. **(H)** The co-localization of NSP6 with Rab9 in HEK293T cells. Cells were transfected with NSP6-RFP along with Rab9-GFP, followed by IF assays at 48 hpt. **(I)** The co-localization of NSP6 with Rab27A and Rab27B in HEK293T cells. Cells were transfected with NSP6-RFP along with Rab27A-GFP or Rab27B-GFP respectively, followed by IF assays at 48 hpt. The nucleus was stained with DAPI (blue). Scale bars represented 5 μm.

**Figure S4**


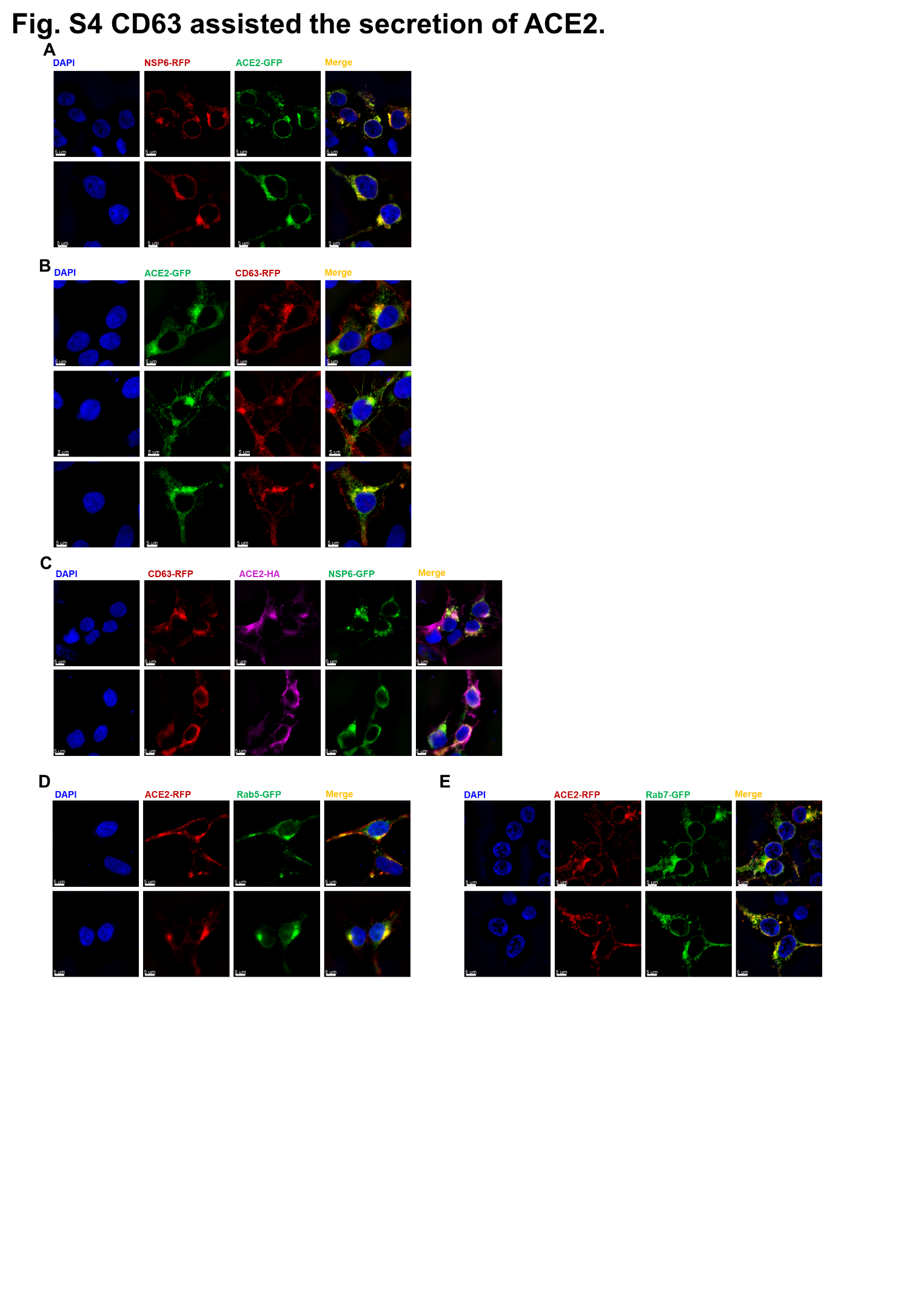


**Figure S4. CD63 assisted the secretion of ACE2.**

**(A)** The co-localization of NSP6 with ACE2 in HEK293T cells. Cells were co-transfected with NSP6-RFP and ACE2-GFP, followed by IF assays at 48 hpt. **(B)** The co-localization of ACE2 with CD63 in HEK293T cells. Cells were co-transfected with ACE2-GFP and CD63-RFP, followed by IF assays at 48 hpt. **(C)** The localization of CD63, ACE2, and NSP6 in HEK293T cells. Cells were co-transfected with CD63-RFP, ACE2-HA, and NSP6-GFP. Samples were subjected to IF assay at 48 hpt. ACE2 proteins were stained with anti-HA antibodies. **(D)** The co-localization of ACE2 with Rab5 in HEK293T cells. Cells were co-transfected with ACE2-RFP along with Rab5-GFP, followed by IF assay at 48 hpt. **(E)** The co-localization of ACE2 with Rab7 in HEK293T cells. Cells were co-transfected with ACE2-RFP along with Rab7-GFP, followed by IF assay at 48 hpt. The nucleus was stained with DAPI (blue). Scale bars represented 5 μm.

**Figure S5**


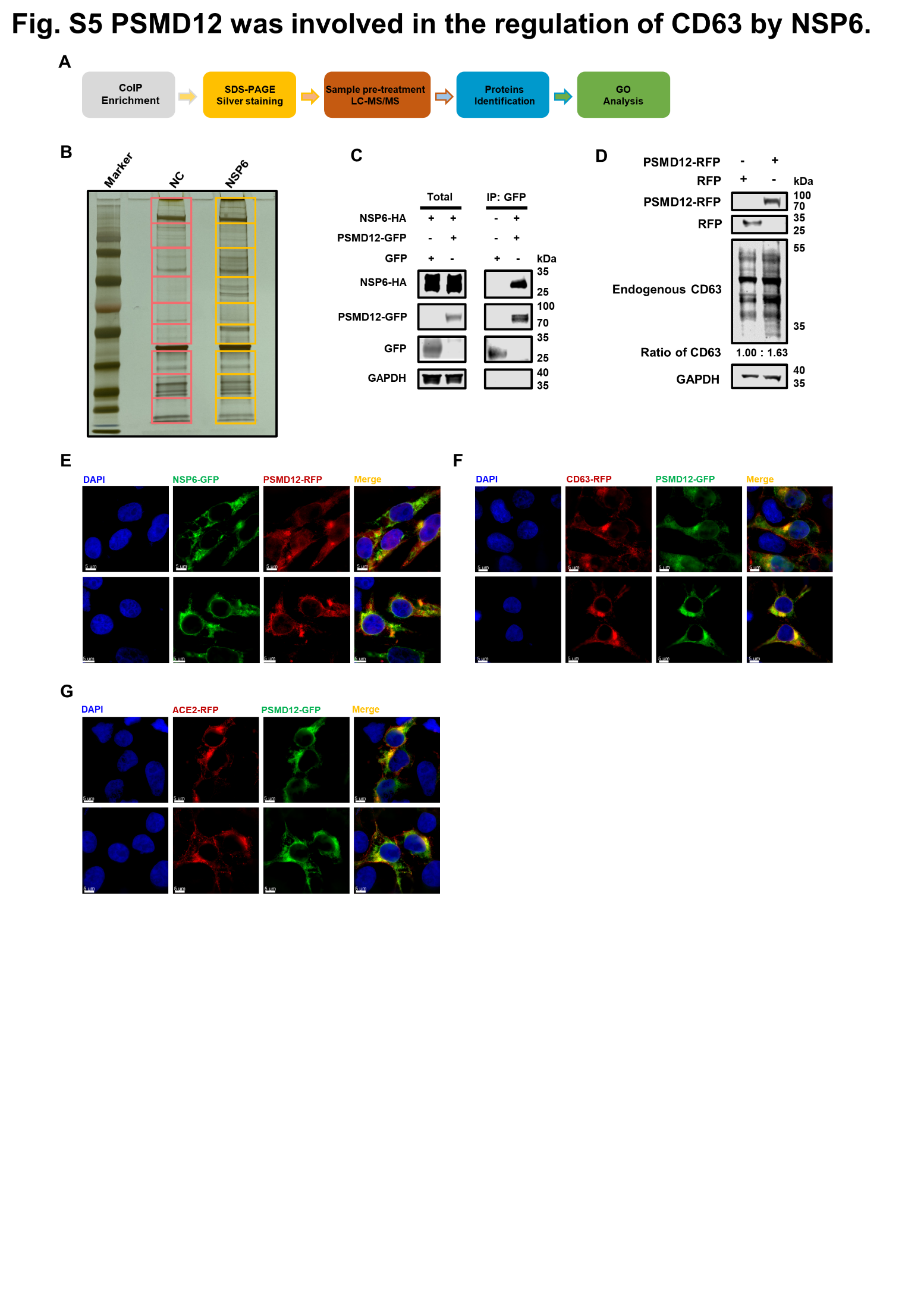


**Figure S5. PSMD12 was involved in the regulation of CD63 by NSP6.**

**(A)** Schematic of CoIP-MS for screening proteins interacted with NSP6. HEK293T cells were overexpressed with GFP (NC group) or GFP-tagged NSP6 (NSP6 group). At 48 h post-transfection, cells were harvested for co-immunoprecipitation to enrich proteins interacting with NSP6. Then, IP samples were subjected to SDS-PAGE and developed with silver staining. Key proteins were identified by LC-MS/MS and analyzed by Gene Ontology (GO) using DAVID Bioinformatics Resources. **(B)** The silver staining image of IP samples. The whole lane of each group was cut into several gel slices (NC group: pink boxes, NSP6 group: yellow boxes). **(C)**The interaction between PSMD12 and NSP6 in HEK293T cells. Cells were transfected with NSP6-HA along with GFP or PSMD12-GFP respectively. Cells were lysed and immunoprecipitated with anti-GFP beads at 48 hpt. **(D)** The effect of PSMD12 on endogenous CD63 was evaluated by western blot in HEK293T cells. HEK293T cells were transfected with RFP or PSMD12-RFP plasmids, followed by western blot against GAPDH (Internal control), RFP, and CD63. **(E-G)** The co-localization of PSMD12 with NSP6, CD63, and ACE2 in HEK293T cells. Cells were co-transfected with PSMD12-RFP and NSP6-GFP (**E**), co-transfected with PSMD12-GFP and CD63-RFP (**F**), or co-transfected with PSMD12-GFP and ACE2-RFP (**G**), followed by IF assay at 48 hpt. The nucleus was stained with DAPI (blue). Scale bars represented 5 μm.

**Figure S6**


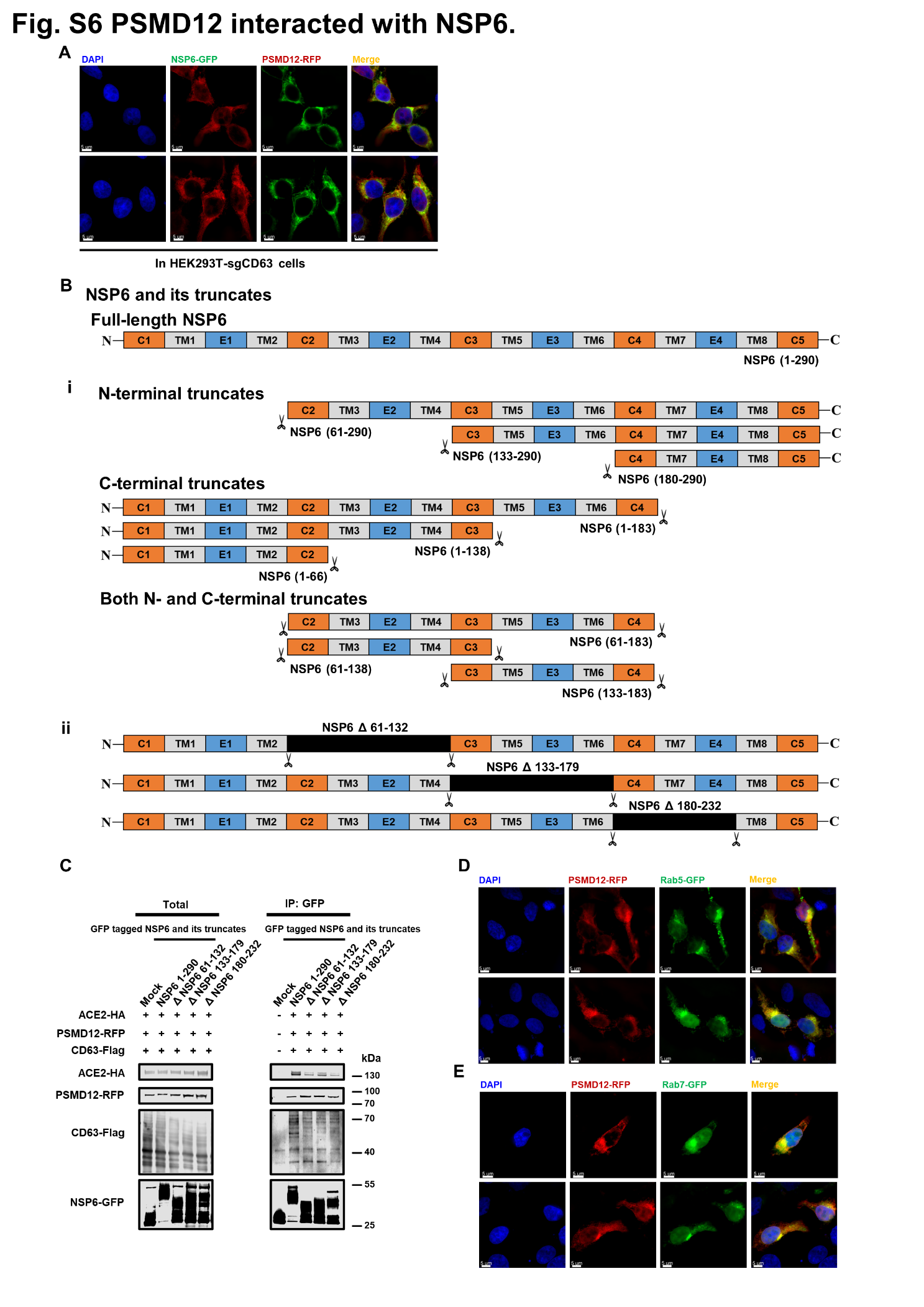


**Figure S6. PSMD12 interacted with NSP6.**

**(A)** The co-localization between NSP6 and PSMD12 in HEK293T-sgCD63 cells. Cells were transfected with NSP6-GFP along with PSMD12-RFP. **(B)** Schematics representation of the full-length NSP6 (1-290 amino acids) and truncated NSP6 used in the study. Extracellular (blue), transmembrane (gray), and cytosolic regions (orange). The cutting sites are indicated by the scissor sign. **(i)** The strategy to generate NSP6 truncates with N- and/or C-terminal deletions from NSP6 1-290. **(i)** The strategy to generate NSP6 truncates that retain the N- and C- terminals while deleting the middle segment of NSP6 1-290. **(C)** The interaction between NSP6 and its mutants (NSP6 Δ61-132, NSP6 Δ133-179, and NSP6 Δ180-232) with CD63, ACE2 and PSMD12 in HEK293T cells. Cells were co-transfected with empty vector (GFP) or GFP-tagged NSP6 and corresponding truncates, along with CD63-Flag, ACE2-HA, and PSMD12-RFP. Cells were lysed and immunoprecipitated with anti-GFP beads at 48 hpt. **(D**-**E)** The co-localization of PSMD12 with Rab5 and Rab7 in HEK293T cells. Cells were transfected with PSMD12-RFP along with Rab5-GFP **(D)**, or transfected with PSMD12-RFP along with Rab7-GFP **(E)**. At 24 hpt, the distributions of these proteins were visualized. The nucleus was stained with DAPI (blue). Scale bars represented 5 μm.

**Figure S7**


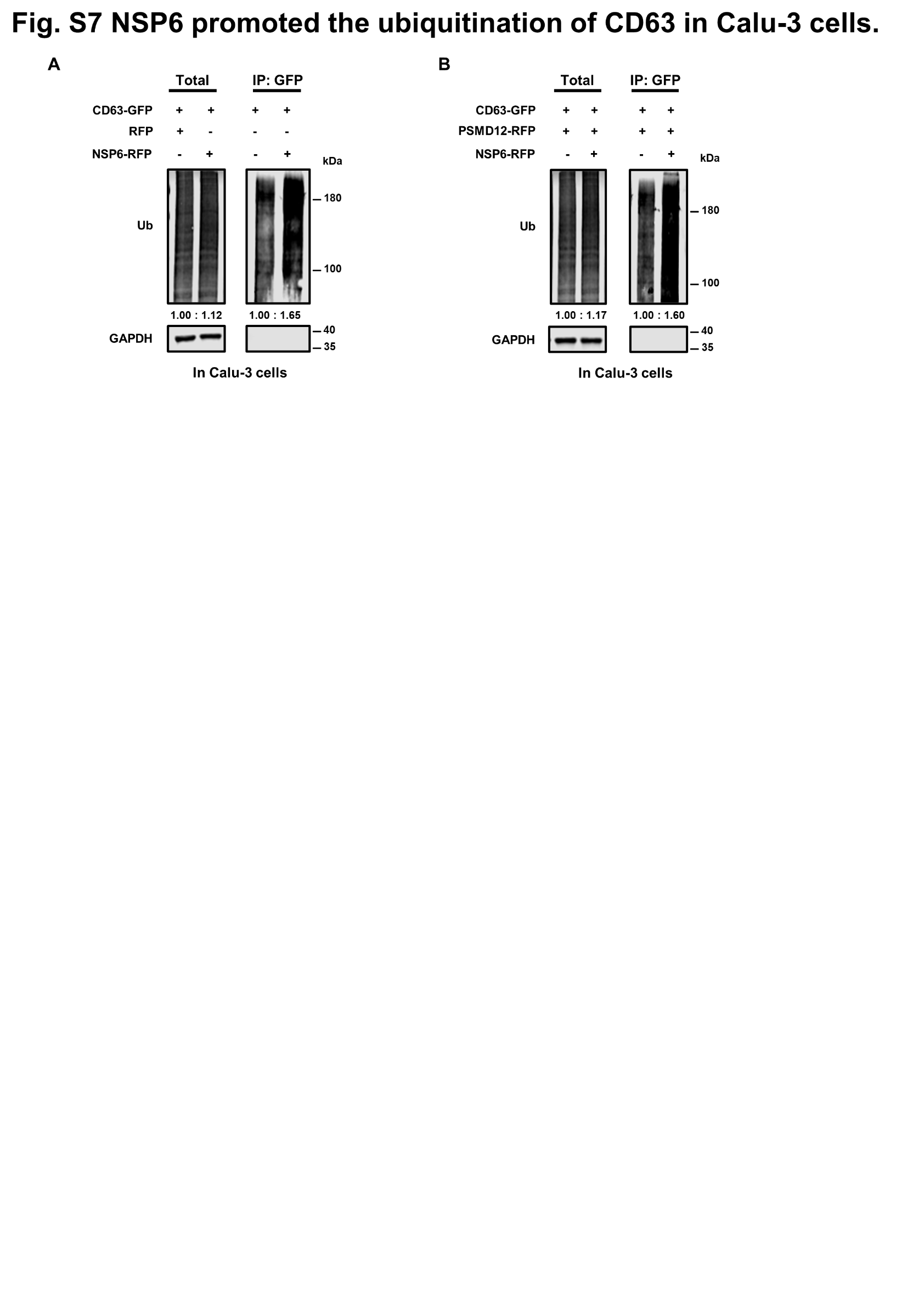


**Figure S7. NSP6 promoted the ubiquitination of CD63 in Calu-3 cells.**

**(A)** NSP6 promoted the ubiquitination of CD63 in Calu-3 cells. Calu-3 cells were transfected with CD63-GFP along with RFP or NSP6-RFP. At 48 hpt, 10 μM MG-132 was added to treat cells for another 12 h. Cells were lysed and immunoprecipitated with anti-GFP beads. The expression levels of ubiquitin-conjugated proteins and GAPDH within both total and IP samples were subjected to western blot assays. **(B)** NSP6 antagonized PSMD12 to enhance the ubiquitination of CD63 in Calu-3 cells. Calu-3 cells were transfected with CD63-GFP and PSMD12-RFP along with empty vector or NSP6-RFP. At 48 hpt, 10 μM MG-132 was added to the culture medium to treat cells for another 12 h. Cells were lysed and immunoprecipitated with anti-GFP beads. The expression levels of ubiquitin-conjugated proteins and GAPDH within both total and IP samples were subjected to western blot assays.
